# Germline VRC01 antibody recognition of a modified clade C HIV-1 envelope trimer and a glycosylated HIV-1 gp120 core

**DOI:** 10.7554/eLife.37688

**Published:** 2018-11-07

**Authors:** Andrew J Borst, Connor E Weidle, Matthew D Gray, Brandon Frenz, Joost Snijder, M Gordon Joyce, Ivelin S Georgiev, Guillaume BE Stewart-Jones, Peter D Kwong, Andrew T McGuire, Frank DiMaio, Leonidas Stamatatos, Marie Pancera, David Veesler

**Affiliations:** 1 Department of BiochemistryUniversity of WashingtonSeattleUnited States; 2 Vaccine and Infectious Disease DivisionFred Hutchinson Cancer Research CenterSeattleUnited States; 3 Vaccine Research CenterNational Institute of Allergy and Infectious Diseases, National Institutes of HealthBethesdaUnited States; 4 Department of Global HealthUniversity of WashingtonSeattleUnited States; California Institute of TechnologyUnited States; Institute of Industrial Science, The University of TokyoJapan

**Keywords:** HIV, HIV envelope, VRC01, cryoEM, crystallography, glycan shield, fusion protein, Virus

## Abstract

VRC01 broadly neutralizing antibodies (bnAbs) target the CD4-binding site (CD4_BS_) of the human immunodeficiency virus-1 (HIV-1) envelope glycoprotein (Env). Unlike mature antibodies, corresponding VRC01 germline precursors poorly bind to Env. Immunogen design has mostly relied on glycan removal from trimeric Env constructs and has had limited success in eliciting mature VRC01 bnAbs. To better understand elicitation of such bnAbs, we characterized the inferred germline precursor of VRC01 in complex with a modified trimeric 426c Env by cryo-electron microscopy and a 426c gp120 core by X-ray crystallography, biolayer interferometry, immunoprecipitation, and glycoproteomics. Our results show VRC01 germline antibodies interacted with a wild-type 426c core lacking variable loops 1–3 in the presence and absence of a glycan at position Asn276, with the latter form binding with higher affinity than the former. Interactions in the presence of an Asn276 oligosaccharide could be enhanced upon carbohydrate shortening, which should be considered for immunogen design.

## Introduction

Despite the tremendous impact of HIV-1 on human health, no efficacious HIV-1 vaccine currently exists. The HIV-1 envelope (Env) glycoprotein is a class-I fusion protein responsible for host attachment and fusion of the viral and cellular membranes (Dalgleish et al., 1984). Following expression, Env trimerizes and undergoes furin-mediated cleavage to yield non-covalent gp120-gp41 pre-fusion trimers anchored in the viral membrane (Haim et al., 2013). As the sole target of neutralizing antibodies, Env is the focus of intense interest for current vaccine design initiatives. However, HIV-1 Env relies on multiple mechanisms of immune evasion – including dense glycosylation, sequence variation, conformational masking, and presentation of decoy epitopes (Burton and Mascola, 2015; Cuevas et al., 2015; Jardine et al., 2015; Kwong et al., 2002; Wei et al., 2003; Zhou et al., 2017). For these reasons, development of an Env-based vaccine capable of eliciting broadly neutralizing antibodies (bnAbs) has proven challenging.

The VRC01-class of bnAbs is of particular interest for HIV-1 vaccine development due to the exceptional potency and breadth of several of its well-characterized members (Huang et al., 2016; Zhou et al., 2015). These bnAbs derive from the VH1-2 variable heavy chain gene (Scheid et al., 2011; Wu et al., 2011), have been isolated from multiple HIV-1-infected patients (Zhou et al., 2013), and putative non-mutated precursors have been identified in naïve individuals (Jardine et al., 2016a). VRC01-class bnAbs are characterized by an unusually short five amino-acid light chain complementary-determining region (CDR) L3 loop (Zhou et al., 2015) and much higher levels of somatic hyper-mutation than antibodies targeting other pathogens (Wu et al., 2015). They bind the CD4-binding site (CD4_BS_) in a way reminiscent of the interactions formed with the viral receptor CD4, making extensive CDRH2-mediated contacts while also exhibiting multiple amino acid alterations in the CDRL1 loop relative to germline precursors (Wu et al., 2015; Zhou et al., 2013). Although *N*-linked glycosylation sites (NLGSs) that surround the CD4_BS_ sterically limit recognition by bnAbs (Zhou et al., 2017), particularly those present at position Asn276 in Loop D and along the V5 loop, mature VRC01 bnAbs overcome this barrier and potently neutralize numerous HIV-1 viral clades (Zhou et al., 2017; Huang et al., 2016; Stewart-Jones et al., 2016; Wu et al., 2015). In contrast, the inferred germline precursors of VRC01-class bnAbs lack detectable binding to trimeric Env constructs harboring glycans at these locations (Jardine et al., 2013; McGuire et al., 2016; McGuire et al., 2013; Medina-Ramírez et al., 2017b; Stamatatos et al., 2017).

Whereas most recombinant trimeric Env antigens do not bind germline precursors of VRC01-class bnAbs, a few recently designed constructs have been shown to bind and activate this specific class of B cell receptors (BCRs) (Jardine et al., 2013; McGuire et al., 2016; McGuire et al., 2013; Medina-Ramírez et al., 2017a). We previously engineered a trimeric HIV-1 Env protein able to bind most VRC01-class precursors (McGuire et al., 2013). This construct was a trimeric gp140 protein derived from the clade C 426c virus and lacked variable loops 1, 2, and 3, along with the putative NLGSs at positions Asn276 (loop D), Asn460, and Asn463 (V5 loop) (McGuire et al., 2016). Other constructs have also been engineered to engage the inferred precursors of VRC01-class bnAbs, all of which harbored mutations eliminating the NLGSs in loop D (at position Asn276) and in the V5 loop (Briney et al., 2016; Jardine et al., 2013; McGuire et al., 2016; McGuire et al., 2013; Medina-Ramírez et al., 2017a; Tian et al., 2016). Additionally, a gp120 core derived from the 01dG5 clade virus, which naturally lacks a glycan at position Asn276, was also shown to engage the inferred germline precursor of the VRC01 antibody (VRC01_GL_) (Wu et al., 2015). Although such glycan-depleted ‘germline-targeting’ immunogens activate B cells expressing germline VRC01-class BCRs in vivo (Briney et al., 2016; Dosenovic et al., 2015; Tian et al., 2016), they largely fail to elicit mature antibodies capable of bypassing the restrictions imposed by the glycan at position Asn276 (Zhou et al., 2017). However, a recent study demonstrated the successful elicitation of CD4_BS_-targeted antibodies, distinct from the VRC01 lineage, upon immunization of rabbits with an engineered clade C Env trimer (Dubrovskaya et al., 2017).

To better understand the potential avenues of elicitation of VRC01-class bnAbs, we structurally characterized complexes between VRC01_GL_ and two clade C Env constructs using a combination of cryo-electron microscopy (cryoEM) and X-ray crystallography. One of the constructs is a soluble trimeric 426c SOSIP with three NLGSs removed at positions Asn276, Asn460, and Asn463, and is based on our prior work (McGuire et al., 2016; McGuire et al., 2013). The second construct is a monomeric 426c core containing all wild-type NLGSs (including those at positions Asn276, Asn460, and Asn463), but lacks variable loops 1, 2, and 3. The 426c strain naturally lacks NLGSs surrounding the CD4_BS_ at positions Asn234 and Asn362(363), which are present in other clades. Our structural analysis revealed that the absence of these glycans leads to a reduction of local oligosaccharide density in the vicinity of the NLGS at position Asn276. Integrating this data with biolayer interferometry (BLI) assays and glycoproteomics, we demonstrate here that VRC01_GL_ could bind to a 426c core construct in the presence of all naturally occurring NLGSs surrounding the CD4_BS,_ including the NLGS at position Asn276 and with its associated glycan. We also show the affinity of VRC01_GL_ for the 426c core could be modulated by altering protein expression conditions to enrich for longer glycans, and also by shortening glycans via endoglycosidase treatment. These results suggest that priming of VRC01-class bnAbs may be possible using an HIV-1 gp120 derivative containing a glycan at position Asn276. Consequently, future epitope-based vaccine design strategies utilizing a 426c core preserving all NLGSs may be a promising route for guiding elicitation of VRC01-class bnAbs.

## Results

### CryoEM structure of VRC01_GL_ in complex with a modified 426c HIV-1 SOSIP glycoprotein trimer

**Figure 1.**
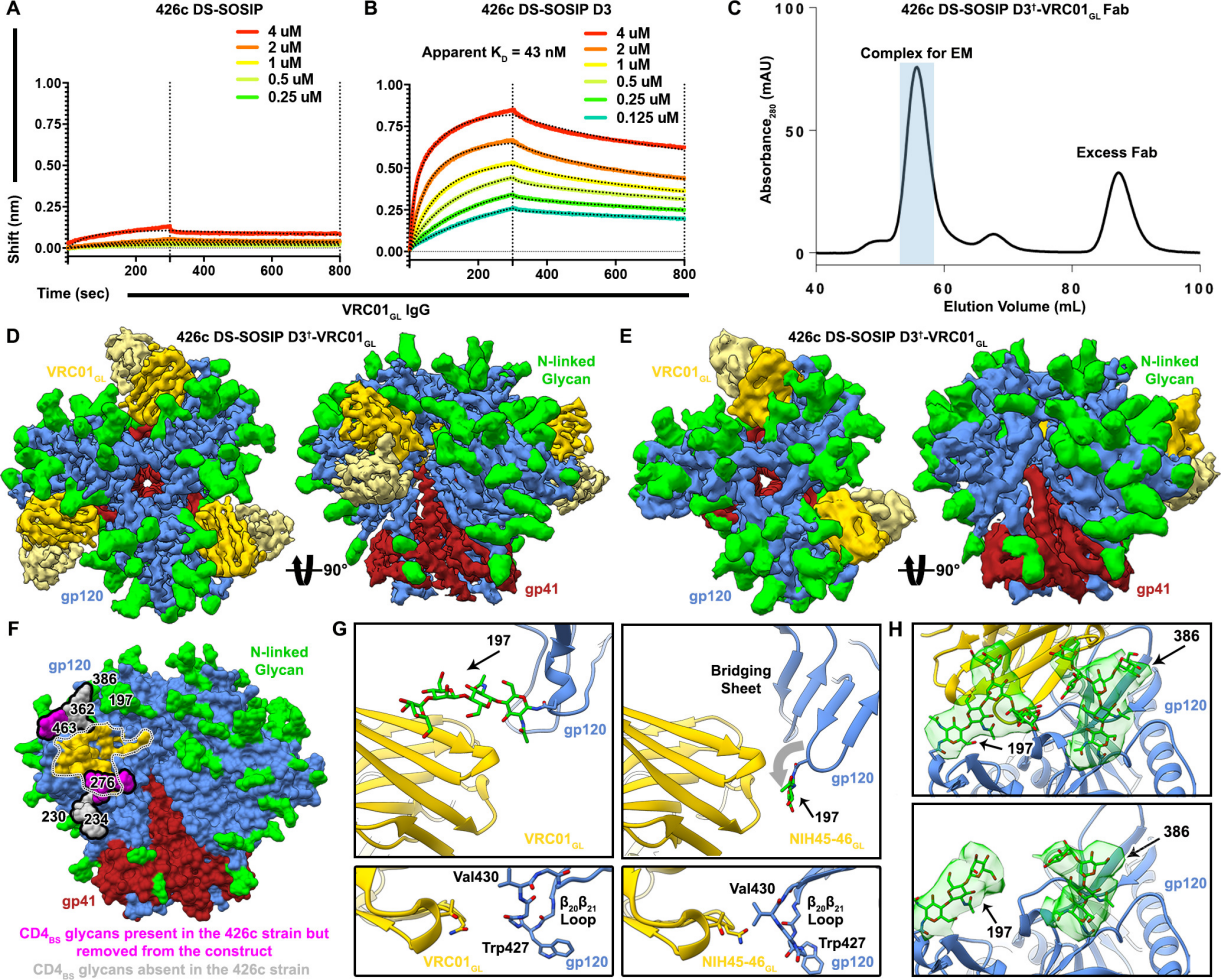
Structural characterization of the 426c DS-SOSIP D3^†^-VRC01_GL_ complex. (**A–B**) BLI binding data of immobilized VRC01_GL_ IgGs binding to WT 426c DS-SOSIP (**A**) or 426c DS-SOSIP D3 trimers. The concentrations of 426c DS-SOSIP trimers injected are indicated on each panel. Fit curves are colored as black dotted lines. A K_D_ could not be determined in (**A**) due to the weak responses observed. The vertical dotted lines indicate the transition between association and dissociation phases. (**C**) Size-exclusion chromatogram of the purified 426c DS-SOSIP D3^†^-VRC01_GL_ complex used for cryoEM structure determination. The pooled fractions used for cryoEM are highlighted in light blue. (**D**) Two orthogonal views of the 3.8 Å cryoEM reconstruction sharpened with a B-factor of −250 Å^2^ whereas the glycan density is shown unsharpened. (**E**) Two orthogonal views of the asymmetric 4.8 Å reconstruction with two bound Fabs. (**F**) Surface representation of the 426c SOSIP trimer highlighting differences in glycosylation compared to the BG505 SOSIP. Glycans not present in 426c are colored light-gray and outlined. Glycans present in the 426c strain but removed by mutation from the 426c DS-SOSIP D3^†^ construct are colored magenta and outlined. The gp120 surface buried at the interface with VRC01_GL_ is indicated as a dotted outline and is colored yellow. (**G**) Comparison of the gp120 bridging sheet conformation when VRC01_GL_-class Fabs are bound to either 426c DS-SOSIP D3^†^ trimer (*Top-left*) or a previously solved 426c gp120 core lacking selected NLGSs, such as the Asn276 NLGS (PDB: 5IGX) (*Top-right*). Comparisons of β_20_β_21_ loop conformations of each complex are shown below corresponding top panels. (**H**) Comparison of glycan density and position between VRC01_GL_-bound and VRC01_GL_-free protomers in the asymmetric cryoEM reconstruction shown in (E). (*Top*) Asn197 and Asn386 glycan density is stronger for protomers bound to VRC01_GL_ Fab than for the gp120 protomer not bound to VRC01_GL_ (*Bottom*). In panels D-H, gp120 protomers are shown in blue, gp41 in red, N-linked glycans in green and VRC01_GL_ in dark and light yellow for the heavy and light chains, respectively.

Based on the known enhanced ability of VRC01_GL_ (and related germline antibodies) to bind 426c constructs lacking putative NLGSs at positions Asn276, Asn460, and Asn463 (McGuire et al., 2013), and the lack of detectable binding to 426c DS-SOSIP (Figure 1A), we engineered a modified 426c DS-SOSIP trimer recapitulating the aforementioned glycan depletion mutations for structural analysis. This construct harbors the S278A, T462A and T465A mutations, abolishing the corresponding NLGSs and enabling binding to VRC01_GL_ (Figure 1B, Figure 1—figure supplement 1). It also contains the SOSIP (Sanders et al., 2002) and the 201C-433C (DS) mutations (Kwon et al., 2015) and is a chimera of 426c gp120 and BG505 gp41 (Joyce et al., 2017). This glycan-depleted protein construct is henceforth referred to as 426c DS-SOSIP D3 (Figure 1—figure supplement 1). The VRC01_GL_ construct comprises the germline VH gene reverted sequences of VH1-2*02, which includes CDRH1 and CDRH2 along with the mature CDRH3 of VRC01 and the germline VK3-11 with the mature CDRL3 of VRC01 (Figure 1—figure supplement 2). Initial complex formation was evaluated using negative-staining EM, which revealed sub-stoichiometric binding of VRC01_GL_ Fab to 426c DS-SOSIP D3 (Figure 1—figure supplement 3A). The VRC01_GL_ Fab appeared to have a much lower affinity for 426c DS-SOSIP D3, compared to the VRC01_GL_ IgG (the latter bound with an apparent equilibrium dissociation constant of 43 nM (Figure 1B), neglecting the effect of avidity). We next attempted to enhance binding of VRC01_GL_ Fab to 426c DS-SOSIP D3 by utilizing a mild glutaraldehyde cross-linking strategy. As expected, we observed significantly increased saturation of 426c DS-SOSIP D3 trimers by VRC01_GL_ Fab, indicating covalent tethering following initial engagement of VRC01_GL_ to the CD4_BS_ was a suitable approach to enrich for and study VRC01_GL_-bound complexes (Figure 1—figure supplement 3A). We therefore engineered a disulfide bond between G459C_gp120_ (426c DS-SOSIP D3^†^) and the VRC01_GL_ heavy chain A60C (denoted as 426c DS-SOSIP D3^†^-VRC01_GL_) (Figure 1—figure supplement 1 and Figure 1—figure supplement 2). This strategy was previously employed to enhance binding of VRC01_MAT_ to SOSIP trimers without altering interface contacts (Stewart-Jones et al., 2016). Using this method, we purified an enriched fraction of Fab-bound trimers and used this sample for structural characterization (Figure 1C, Figure 1—figure supplement 3A–D). This strategy led us to determine two cryoEM reconstructions of the 426c DS-SOSIP D3^†^-VRC01_GL_ complex (Figure 1D–E, Figure 1—figure supplement 3E–H, Figure 1—figure supplement 4A–D, Figure 1—figure supplement 5): one with three bound Fabs at 3.8 Å resolution (Figure 1D, Table 1, Figure 1—figure supplement 3E, Figure 1—figure supplement 4A–B), and one with two bound Fabs at 4.8 Å resolution (Figure 1E, Figure 1—figure supplement 3H, Figure 1—figure supplement 4C–D).

**Figure 1—figure supplement 1.**
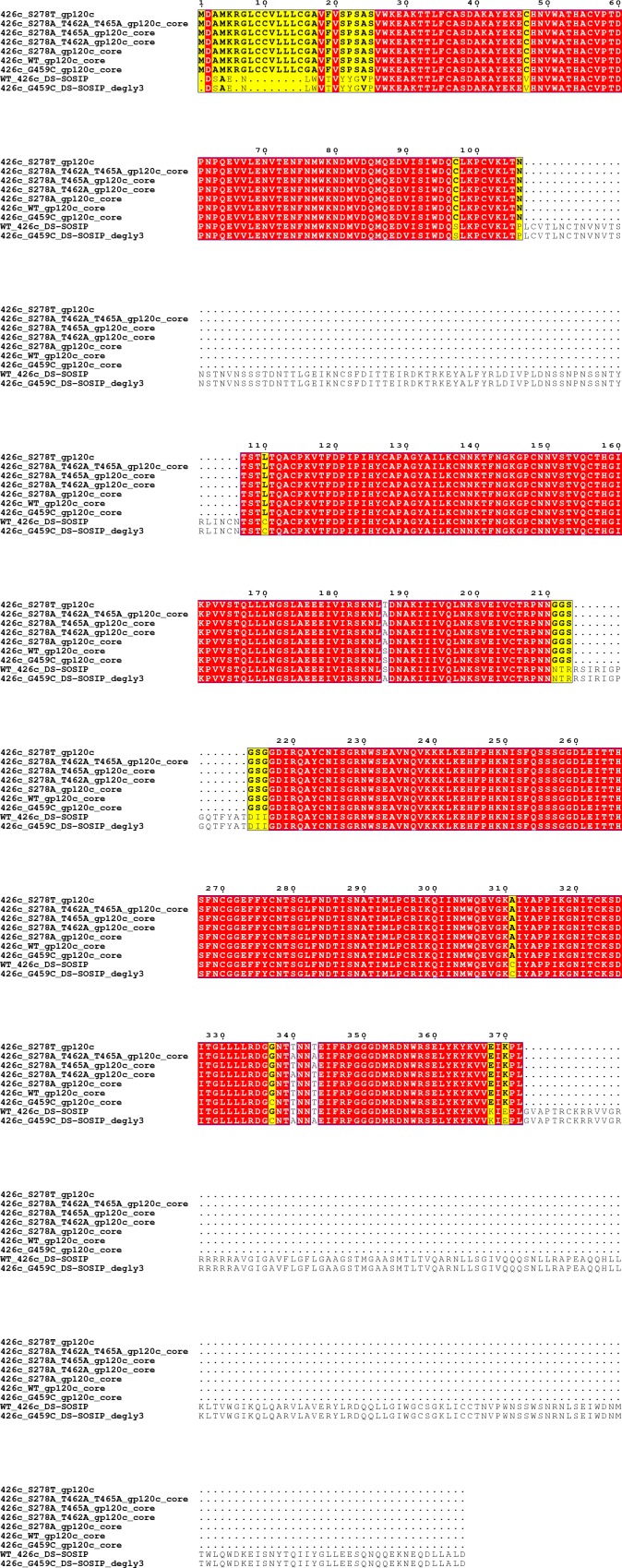
Multiple sequence alignment of analyzed HIV-1 426c constructs. HIV-1 constructs derived from the 426c strain used in this study were subjected to multiple sequence alignment using Clustal Omega and rendered using ESPript (Gouet et al., 1999; Sievers and Higgins, 2014). Residues highlighted in red signify identical amino acids conserved across all aligned constructs. Similar residues are highlighted in bold and colored yellow.

**Figure 1—figure supplement 2.**
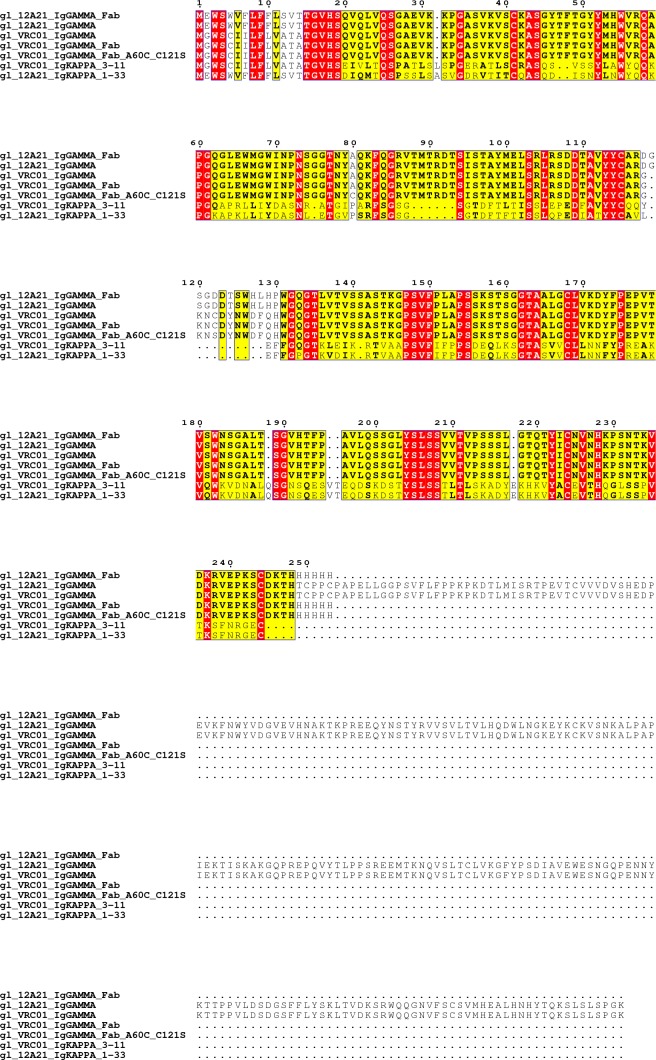
Multiple sequence alignment of analyzed antibody and Fab constructs. VRC01_GL_-class antibody and Fab constructs used in this study were subjected to multiple sequence alignment using Clustal Omega and rendered using ESPript (Gouet et al., 1999; Sievers and Higgins, 2014). Residues highlighted in red signify identical amino acids conserved across all aligned constructs. Similar residues are highlighted in bold and colored yellow.

**Figure 1—figure supplement 3.**
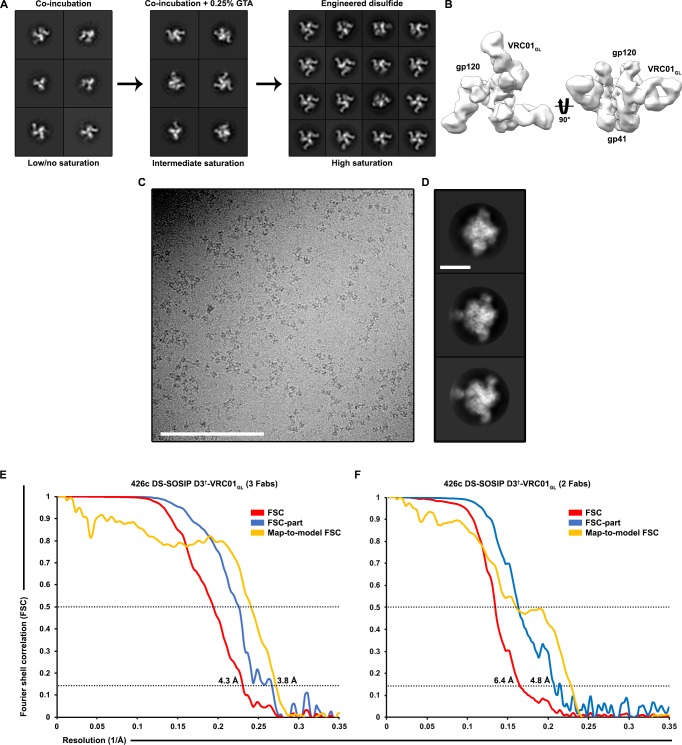
Structural characterization of the 426c DS-SOSIP D3^†^-VRC01_GL_ complex. (**A**) Strategy implemented to increase the occupancy of VRC01_GL_ Fab for structural studies. VRC01_GL_ can engage 426c DS-SOSIP D3, but its low apparent affinity relative to VRC01_GL_ IgG precludes saturation at the concentrations used for negative staining EM imaging (*Left*). Mild glutaraldehyde (GTA) crosslinking (0.25% GTA for 45 s followed by quenching with 1M Tris) increased VRC01_GL_ saturation of 426c DS-SOSIP D3 (*Middle*). Engineering a disulfide bond between 426c DS-SOSIP D3 (G459C_gp120_) and the heavy chain of VRC01_GL_ (A60C_HC_) further increased gp120 saturation of the trimer (*Right*). (**B**) 3D reconstruction of negatively stained 426c DS-SOSIP D3^†^-VRC01_GL_. (**C–D**) Representative micrograph (**E**) and 2D class averages (**F**) of frozen-hydrated 426c DS-SOSIP D3^†^-VRC01_GL_. Scale bars represent 200 nm (**E**) or 200 Å (**F**). (**E**) Fourier shell correlation (FSC) curves of the 426c DS-SOSIP D3^†^-VRC01_GL_ complex with three Fabs bound showing an estimated resolution of 3.8 Å. (**F**) Fourier shell correlation curves of the 426c DS-SOSIP D3^†^-VRC01_GL_ complex with two Fabs bound showing an estimated resolution of 4.8 Å. The top and bottom horizontal dashed lines show the 0.5 and 0.143 cutoffs used for resolution estimates for map-to-model or gold-standard FSC, respectively. Both conventional FSC and FSC-part, as reported in Frealign, are shown.

**Figure 1—figure supplement 4.**
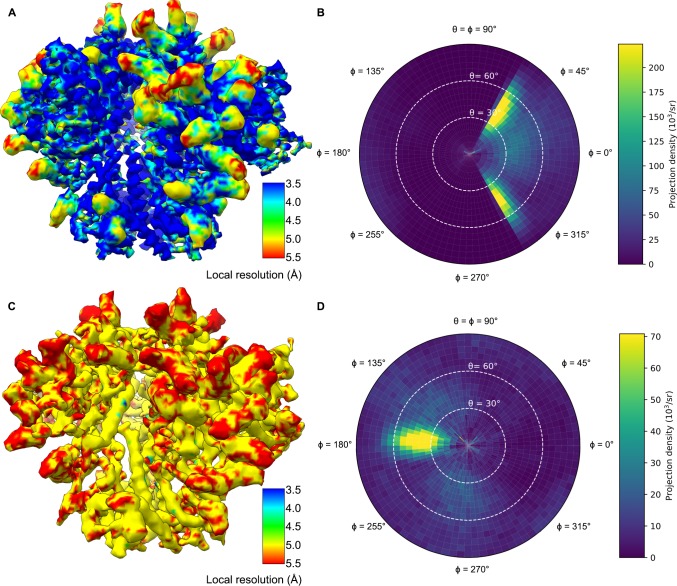
Validation of the 426c DS-SOSIP D3^†^-VRC01_GL_ cryoEM reconstructions. (**A**) Local resolution estimates of 426c DS-SOSIP D3^†^ bound to three copies of VRC01_GL_-A60C_HC_ as determined using ResMap (Kucukelbir et al., 2014). (**B**) Graphical plot depicting distribution of particle image orientations. (**C**) Local resolution estimates of 426c DS-SOSIP D3^†^ bound to two copies of VRC01_GL_-A60C_HC_ as determined using ResMap(Kucukelbir et al., 2014). (**D**) Graphical plot depicting distribution of particle image orientations.

**Figure 1—figure supplement 5.**
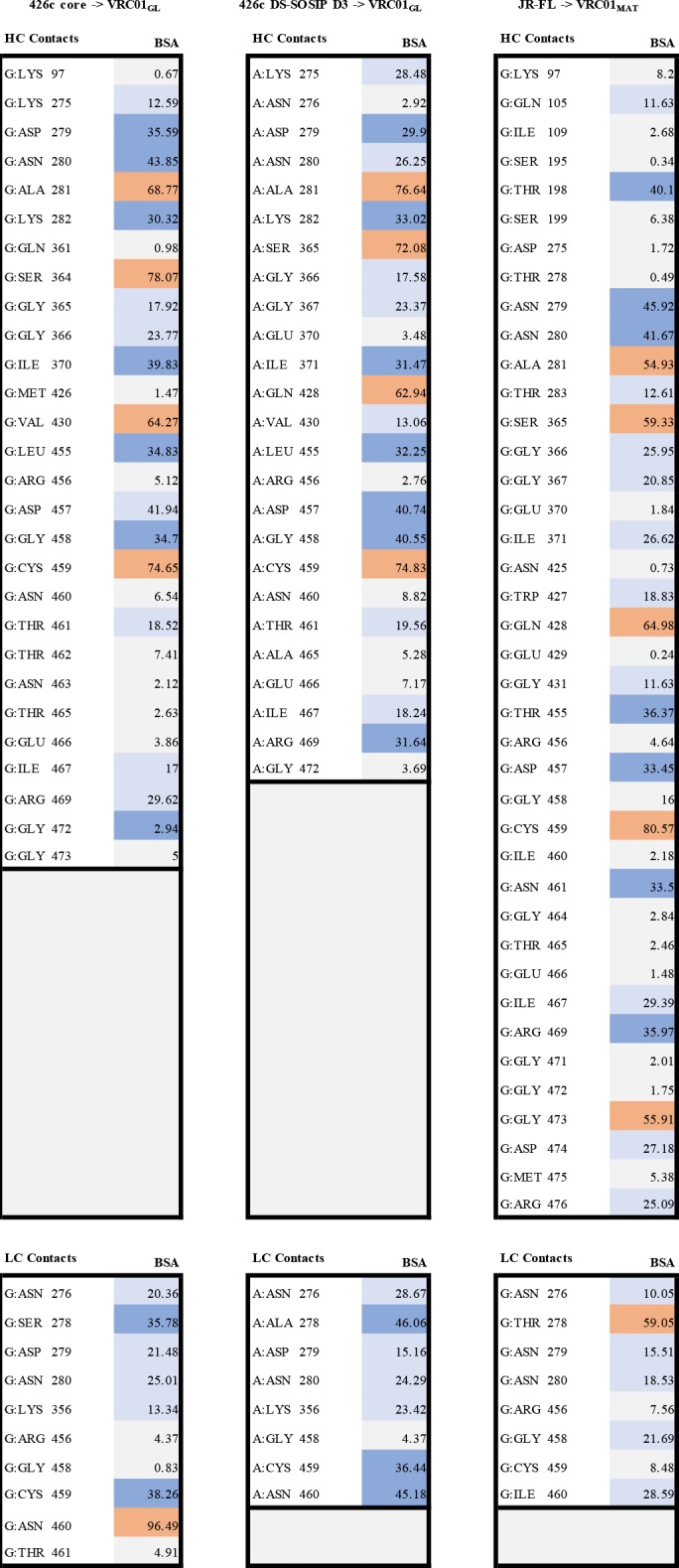
Comparison of gp120 interface contacts between VRC01_GL_ and VRC01_MAT_. Tables highlighting gp120 residues and their associated buried surface area (BSA) which comprise the interface with VRC01-class antibodies, as determined by PISA. BSA values are colored light gray for values ranging between 0.1 and 10.0, light blue for values between 10.1 and 30.0, dark blue for values between 30.1 and 50.0, and red for values > 50.0 Å^2^. HC: heavy chain; LC: light chain.

**Figure 1—figure supplement 6.**
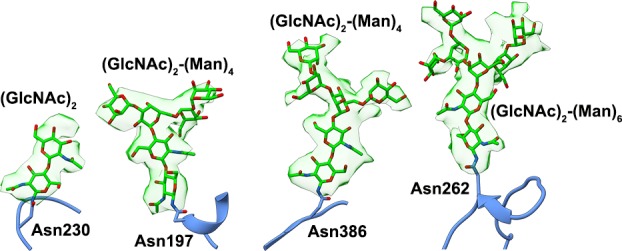
Example of glycans resolved in the 426c DS-SOSIP D3^†^-VRC01_GL_ structure. CryoEM density (green semi-transparent surface) and atomic model for glycans at positions Asn230, Asn197, Asn386 and Asn262 are shown.

**Table 1.** CryoEM data collection, refinement, and model validation statistics.

Parameter	Value
Data Collection	
No. of Micrographs	1993
No. of Particles	134,443
Pixel size, Å	1.36
Defocus range, μM	2.0–3.5
Voltage, kV	300
Dose Rate, counts/pix/sec	8
Electron dose, e^-^/Å^2^	43
Refinement	
Resolution, Å	3.8
Map-sharpening B factor, Å^2^	−230
Model validation (3 Fab structure)	
Favored rotamers, %	98.36%
Poor rotamers, %	0.30%
Ramachandran outliers, %	0.13%
Clash Score	0.99
Molprobity score EM ringer score	1.02 1.97

Similarly to what was reported for the B41 SOSIP trimer (Ozorowski et al., 2017), the 426c DS-SOSIP D3^†^-VRC01_GL_ V1/V2 apex is closed under cryoEM conditions (Figure 1D,E,F) whereas it appears open in the conditions we used for negative-staining sample preparation (Ozorowski et al., 2017) (Figure 1—figure supplement 3B). We note that the closed 426c DS-SOSIP D3^†^-VRC01_GL_ trimeric Env conformation observed in cryoEM lacks a formed gp120 bridging sheet, which is reminiscent of other closed SOSIP trimer structures (Figure 1G) (Julien et al., 2015; Lyumkis et al., 2013b; Pancera et al., 2014; Stewart-Jones et al., 2016). VRC01_GL_-class antibodies have recently been shown to also bind to core gp120 constructs in the presence of a bridging sheet (Scharf et al., 2016). Our data reveal that VRC01_GL_ could also bind a prefusion closed conformation, which had previously only been reported for its mature counterpart, VRC01_MAT _(Stewart-Jones et al., 2016) (Figure 1G).

### Structural analysis of the region surrounding the CD4_BS_ in 426c DS-SOSIP D3

Removal of CD4_BS_-surrounding carbohydrates has been shown to enhance binding of CD4_BS_-targeted germline VRC01-class antibodies and to increase the antigenicity of this region (McGuire et al., 2016; McGuire et al., 2013; Stamatatos et al., 2017; Zhou et al., 2017). Our structural analysis reveals that the 426c DS-SOSIP naturally lacks an NLGS at position Asn234 near the CD4_BS_, which is otherwise conserved in 80% of known circulating HIV-1 strains (Crooks et al., 2015). Instead, 426c features a glycan at Asn230 that is more remote from the VRC01 epitope than glycan Asn234 (Jardine et al., 2016b) (Figure 1F). The oligosaccharide at position Asn230 appears to be highly dynamic, since only the two proximal N-acetyl-glucosamine (GlcNAc) moieties are resolved in the reconstruction (Figure 1—figure supplement 6) and does not interact with VRC01_GL_ or other glycans in the complex. Previous structural characterization of clades A and G SOSIP trimers established that glycans at positions Asn276 and Asn234 are in close proximity to each other and likely restrain each others’ conformational freedom (Jardine et al., 2016b; Stewart-Jones et al., 2016; Zhou et al., 2017). The absence of glycan Asn234 in 426c gp120 reduces local carbohydrate crowding near the CD4_BS _which could increase accessibility of this neutralization supersite (Stewart-Jones et al., 2016) and lead to altered local glycan processing (Behrens et al., 2018; Bonomelli et al., 2011).

The 426c DS-SOSIP also lacks an NLGS at position Asn362(363), which is present in 42% of strains deposited in the HIV database (Gaschen et al., 2001) (Figure 1F). This oligosaccharide is located distally from the viral membrane side of the SOSIP trimer (Figure 1F) and is sandwiched between the VRC01_MAT_ heavy chain and glycan Asn386 in the structure of VRC01_MAT_ bound to the JR-FL SOSIP trimer (clade B) (Stewart-Jones et al., 2016). Analysis of the asymmetric 426c DS-SOSIP D3^†^-VRC01_GL_ structure, comprising two Fabs, revealed that Fab-bound protomers feature slightly better-resolved density for glycan Asn386 than the free protomer when visualized at the same contour level (Figure 1H). These observations suggest that VRC01_GL_ may stabilize the Asn386 glycan either through reduction of its conformational freedom and/or via direct interactions with the Fab framework region. The absence of glycan Asn362 or other topologically equivalent oligosaccharides in the 426c gp120 sequence likely contributes to increased accessibility of the CD4_BS_ to VRC01_GL_-class bnAbs due to the close proximity of this glycan to the epitope (Stewart-Jones et al., 2016) (Figure 1F).

Similarly to what is observed in available VRC01_MAT_/SOSIP complex structures (Stewart-Jones et al., 2016), glycan Asn197 density is also strongest when bound to VRC01_GL_, but appears weaker in the unbound protomer (Figure 1H), again indicating either Fab-induced stabilization or restriction of movement. The position of glycan Asn197 differs substantially between available structures of monomeric gp120 constructs bound to VRC01_GL_-class Fabs and the VRC01_GL_-bound SOSIP trimer reported here (Figure 1G) (Scharf et al., 2016). This variation in Asn197 positioning is guided by the formation of the gp120 bridging sheet in monomeric gp120, which would otherwise only form following CD4 receptor binding in the context of trimeric Env (Figure 1G) (Kwon et al., 2012; Zhou et al., 2010). This conformational difference includes the β_20_/β_21_ loop, whose orientation in the 426c DS-SOSIP D3^†^-VRC01_GL_ complex differs relative to crystal structures of VRC01_GL_-class antibodies in complex with monomeric gp120 (Figure 1G) (Scharf et al., 2016). Although the β_20_/β_21_ loop is close to the VRC01 paratope, VRC01_MAT_ was reported to have minimal preference in the conformation of the bridging sheet or β_20_/β_21_ region, as 87% of its contact surface area includes the conformationally invariant outer domain of gp120 (Zhou et al., 2010). Whether or not the conformation of the β_20_/β_21_ region directly impacts germline VRC01-class antibody binding affinities remains unclear. However, VRC01_GL_ in complex with gp120 constructs lacking this domain have been determined (eOD-GT6 and eOD-GT8), demonstrating that these germline mAbs do not strictly require this region for CD4_BS_ recognition when glycans surrounding the CD4_BS_ are also removed (Jardine et al., 2013).

### Wild-type V5 loop NLGSs of the 426c core did not hinder binding to VRC01_GL_ Fabs

**Figure 2.**
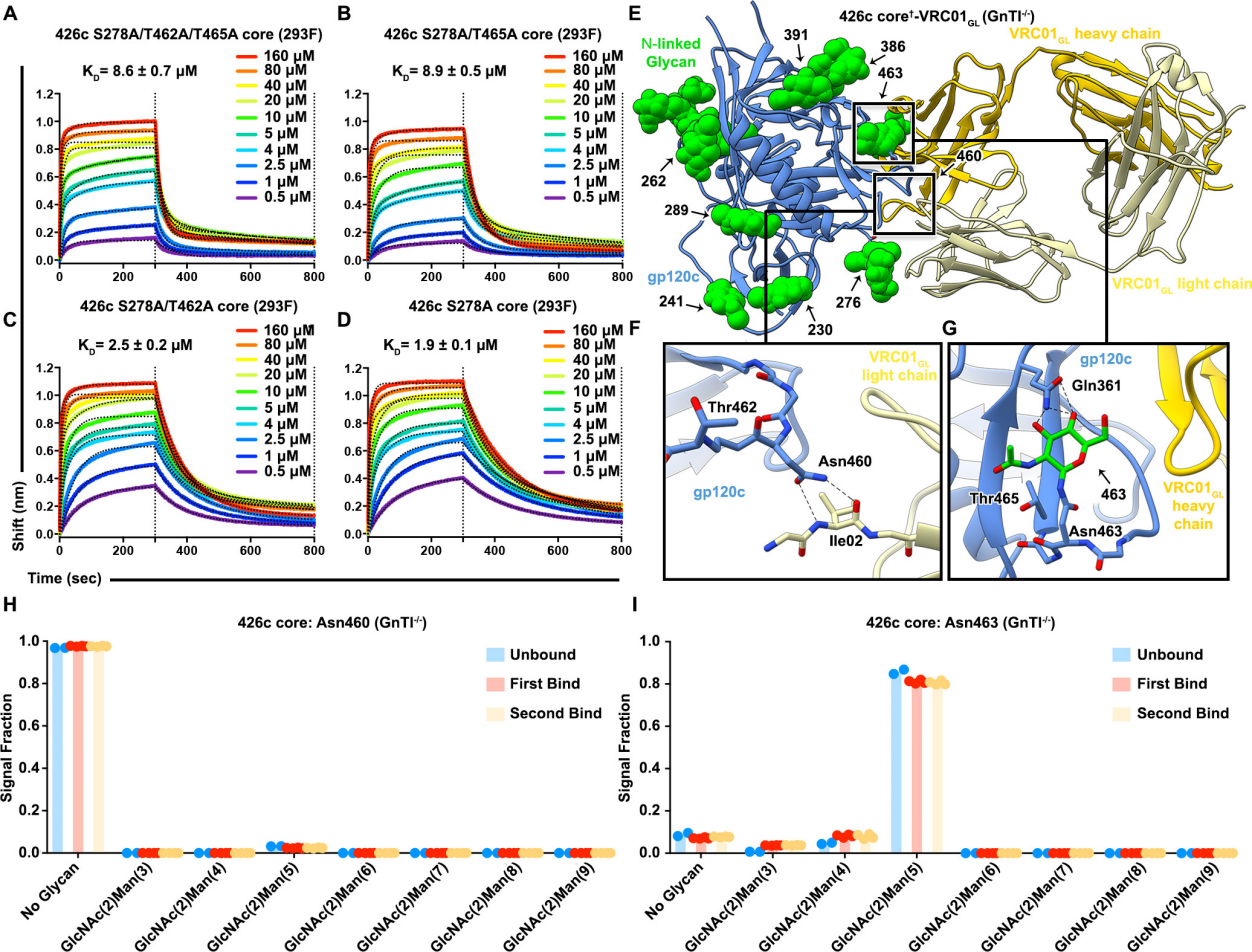
Reintroduction of V5 loop NLGSs does not hinder VRC01_GL_ binding to the 426c core. (**A–D**) BLI curves and the corresponding equilibrium dissociation constants for VRC01_GL_ IgG binding to the S278A/T462A/T465A (**A**), S278A/T462A (**B**), S278A/T465A (**C**), and S278A (**D**) 426c core constructs lacking either one or several glycans in the V5 and D loops. The concentrations of 426c core injected and the color key is indicated on each panel. Fitted curves are colored as black dotted lines. The vertical dashed lines indicate the transition between association and dissociation phases. (**E**) Ribbon diagram of the 426c core^†^-VRC01_GL_ complex crystal structure. gp120 is colored blue, VRC01_GL_ Fab is colored yellow (heavy chain: dark yellow; light chain: light yellow), and resolved gp120 glycans are shown in surface representation and colored green. (**F**) Close-up view of the gp120 Asn460 contacts with the backbone carbonyl and amide groups of the light chain VRC01_GL_ residue Ile02. (**G**) Close-up view of the (GlcNAc)_1_ at position Asn463 of gp120. Oligosaccharides are labeled by the corresponding Asn residue they are linked to. Hydrogen bonds are represented as dashed lines. (**H–I**) Semi-quantitative LC-MS/MS analysis of VRC01_GL_-based IP experiments depicting the relative signal intensities for identified Asn460 (**H**) and Asn463 (**I**) glycoforms in unbound (blue), first binding event (red), and second binding event (yellow) fractions. The ‘unbound’ material indicates 426c core glycoforms that did not bind VRC01_GL_ well following three binding steps. The ‘first’ binding event corresponds to 426c core elution fractions following collection of the sample flow-through and three rigorous wash cycles. The ‘second’ binding event follows a rebinding of the aforementioned flow-through, performing three additional washes, and eluting any residual bound material from the VRC01_GL_ affinity column and collecting this fraction. Colored dots associated with their corresponding histogram bars represent individual values extracted from each experimental replicate, with the bar itself representing the experimental mean signal fraction.

One of the mechanisms by which HIV-1 Env has evolved to avoid detection by the progenitors of VRC01-class bnAbs is by selection of V5 loop NLGSs (Huang et al., 2016; Li et al., 2011; Zhou et al., 2010) which sterically limit access to the CD4_BS_. The observation that VRC01_GL_ may accommodate carbohydrates surrounding the CD4_BS_ in our 426c DS-SOSIP D3^†^-VRC01_GL_ cryoEM structure prompted us to assess the effect on binding of the two V5 loop putative NLGSs mutated in 426c DS-SOSIP D3^†^-VRC01_GL_. With 426c Env trimers, we previously found that VRC01_GL_ binding could be detected following removal of glycan Asn276, and was further enhanced following removal of wild-type NLGSs at positions Asn460 and Asn463 (McGuire et al., 2013). We also demonstrated that removing the V1/V2, and V3 loops in gp140 Env trimers further increased binding of multiple VRC01-class germline antibodies relative to trimers only containing glycan-depleting mutations (McGuire et al., 2014; McGuire et al., 2016). However, the effects of V1/V2 and V3 loop deletion on VRC01_GL_ binding to 426c core constructs in the presence of glycans remains unclear. Here we reintroduced the two NLGSs at positions Asn460 and Asn463 (in the V5 loop) and assessed their individual and cumulative effects on VRC01_GL_ engagement of the 426c core construct comprised of gp120 residues 44 to 492, and lacking the V1/V2 and V3 variable loops (Kwon et al., 2012). Expanding on our prior work (McGuire et al., 2016), the following four 426c core glycan-deleted combinations were tested using HEK293F-expressed protein constructs: S278A/T462A/T465A, S278A/T465A, S278A/T462A, and S278A (Figure 1—figure supplement 1, Figure 2A–D, Supplementary file 1).

**Figure 2—figure supplement 1.**
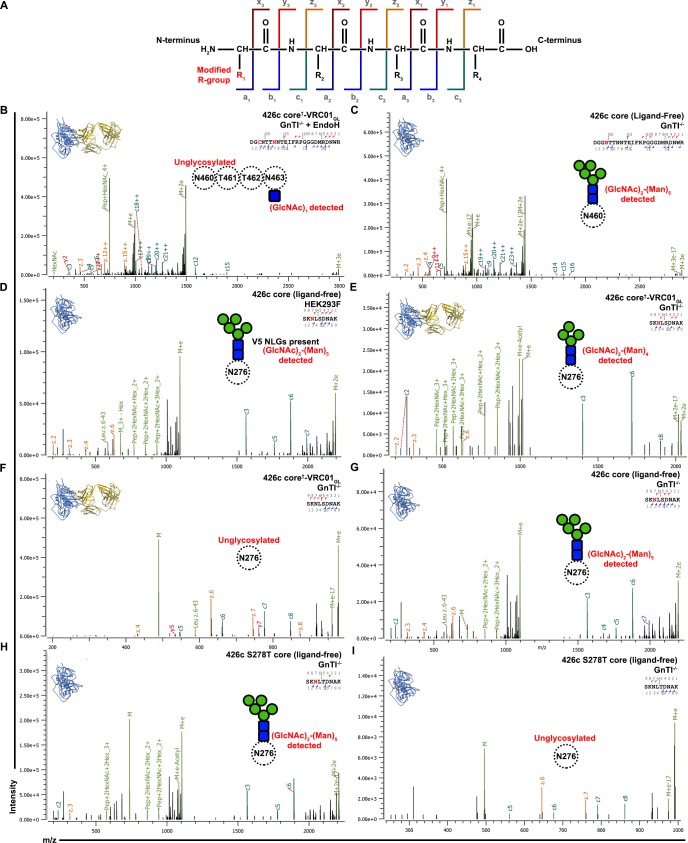
Representative LC-MS/MS glycan identifications of 426c core constructs. The top-left ribbon diagram corresponds to the sample analyzed. The LC-MS/MS fragmentation pattern is indicated in the top-right inset. A graphical depiction of the Asn276 residue (dotted white circle) and its associated identified glycan (blue: N-acetyl glucosamine, green: mannose) are represented on the spectrum. Green peak labels correspond to precursor peptides with/without LC-MS/MS fragmentation occurring within the glycan. Red/orange peak labels represent identified x, y, and z fragments. Blue/teal peak labels highlight identified a, b, and c fragments. **A**) Schematic of possible LC-MS/MS peptide fragmentation patterns. The peptide N- and C-termini are labeled. Possible fragmentation positions are denoted as grey text, with x, y, and z fragments represented as red/orange labels and blue/teal fragments representing a, b, and c fragments. Modified R-groups are denoted in red text. (**B–C**) Representative LC-MS/MS spectra of detected V5 glycosylation profile of the 426c core^†^-VRC01_GL_ (GnTI^-/-^-expressed) (**B**) and a ligand-free 426c core containing a glycan at position Asn460 (GnTI^-/-^-expressed) (**C**). **D–J**) Various additional representative LC-MS/MS spectra of an Asn276 glycopeptide containing a (GlcNAc)_2_-(Man)_5_ oligosaccharide from ligand-free 426c core (HEK293F-expressed) (**D**), an Asn276 glycopeptide of the 426c core^†^-VRC01_GL_ complex with a detectable (GlcNAc)_2_-(Man)_4_ sugar (GnTI^-/-^-expressed) (**E**), an Asn276 peptide from the 426c core^†^-VRC01_GL_ complex that is unglycosylated at position Asn276 (GnTI^-/-^-expressed) (**F**), a 426c core Asn276 glycopeptide containing a (GlcNAc)_2_-(Man)_5_ oligosaccharide (GnTI^-/-^-expressed) (**G**), a 426c S278T core Asn276 glycopeptide containing a (GlcNAc)_2_-(Man)_5_ oligosaccharide (GnTI^-/-^-expressed) (**H**), and an Asn276 peptide of a 426c S278T core that is unglycosylated at position Asn276 (GnTI^-/-^-expressed) (**I**).

Reintroduction of the NLGS at position Asn460 (S278A/T465A) in the 426c core had no detectable impact on VRC01_GL_ binding affinity despite the predicted overlap of a putative carbohydrate at position Asn460 with the bound VRC01_GL_ Fab (Guo et al., 2012) (Figure 2A–B, Supplementary file 1). Indeed, our previous work removing NLGSs at positions Asn460 and Asn463 via the N460D/N463D mutations had only a relatively minor impact on VRC01_GL_ engagement to trimeric 426c constructs compared to the large increase in binding observed following removal of the native Asn276 NLGS (McGuire et al., 2016; McGuire et al., 2013). To better understand the molecular rationale of these observations with the monomeric 426c core construct, we engineered a disulfide-linked 426c core^†^-VRC01_GL_ complex containing all wild-type NLGSs (426c core^†^-VRC01_GL_). We co-expressed these proteins using HEK293 GnTI^-/-^ cells, which lack N-acetyl-glucosaminyltransferase I activity and thus are unable to generate complex *N*-linked carbohydrates (Wright and Morrison, 1994). We then determined its crystal structure at 2.3 Å resolution after endoglycosidase H (EndoH) treatment to facilitate crystallization (Depetris et al., 2012; Freeze and Kranz, 2010) (Figure 2E, Table 2). Despite harboring an NLGS, no glycan density could be resolved at position Asn460 in either of the two molecules of the asymmetric unit. Instead, the Asn460 side chain is hydrogen bonded to the backbone amide and carbonyl groups of the VRC01_GL_ light-chain residue, Ile02 (Ile02_LC_) (Kong et al., 2016)(Figure 2F). In support of this observation, we detected only unglycosylated Asn460 peptide fragments when analyzing tryptic digests of this sample with liquid chromatography coupled to electron transfer/high-energy collision-dissociation tandem mass-spectrometry (LC-MS/MS) (Figure 2H, Figure 2—figure supplement 1A,B). Furthermore, only low levels of glycosylation were detected at Asn460 by qualitative LC-MS/MS analysis of unliganded 426c core (lacking the G459C mutation) (Figure 2—figure supplement 1C). Additionally, we performed VRC01_GL_-based immunoprecipitation (IP) experiments utilizing a VRC01_GL_ affinity column and the same 426c core construct. Semi-quantitative LC-MS/MS comparison of the 426c gp120 core samples from fractions that did not bind to VRC01_GL_ (‘unbound’ flow-through), and those that did (‘bound’ elution), revealed no difference in glycan occupancy of the Asn460 NLGS (Figure 2H). This indicated this sequon is rarely glycosylated in 426c core and explains its negligible impact on VRC01_GL_ binding. The predicted overlap of glycan Asn460 with VRC01_GL_ and the absence of a resolved proximal GlcNAc in the crystal structure of 426c core^†^-VRC01_GL_ also suggests a likely strict preference for the unglycosylated Asn460 glycoform of the 426c gp120 for binding (Figure 2F).

**Table 2.** Crystallographic data collection and refinement statistics

	426c core^†^-VRC01_GL_
**Data collection**	
Space group	C2
Cell dimensions	
* a*, *b*, *c* (Å)	197.082, 109.003, 103.225
*α*, *β*, *γ* (°)	90.000, 114.468, 90.000
Resolution (Å)	50–2.32 (2.36–2.32)*
*R*_sym_ or *R*_merge_	0.076 (0.643)*
*I*/s*I*	23.4 (1.8)*
Completeness (%)	95.6 (66.8)*
Redundancy	7.4 (5.7)*
CC1/2	(0.823)*
**Refinement**	
Resolution (Å)	46.98–2.315 (2.398–2.315)*
No. reflections	83086
*R*_work_/*R*_free_	24.38/29.55 (42.67/49.28)
No. atoms	12470
Protein	11746
Water	325
Ligand	399
B-factors (Å^2^)	74.22
Protein	73.57
Water	69.62
Ligand	97.10
R.m.s deviations	
Bond lengths (Å)	0.003
Bond angles (°)	0.60
Ramachadran Favored %	93.39
Ramachadran Outliers %	0.13
MolProbity all-atoms clashscore	4.05

We furthermore observed that reintroduction of the Asn463 NLGS (S278A/T462A or S278A) also did not result in a reduction in VRC01_GL_ binding relative to the 426c S278A/T462A/T465A core (Figure 2A,C,D, Supplementary file 1). This result was unexpected, as V5 glycosylation of Env trimers containing all variable loops have been reported to negatively affect VRC01_GL_ recognition of the CD4_BS_ (Huang et al., 2016; Li et al., 2011; McGuire et al., 2013; Zhou et al., 2010). We observed electron density for the proximal GlcNAc linked to Asn463 in one of the two molecules of the asymmetric unit of the 426c core^†^-VRC01_GL_ crystal structure and cross-validated the presence of this post-translational modification using LC-MS/MS (Figure 2G). VRC01_GL_-based IP experiments followed by semi-quantitative LC-MS/MS validated these structural observations by detecting an Asn463 glycosylation profile which was indistinguishable between ‘bound’ elution and ‘unbound’ flow-through fractions (Figure 2I). This supports our BLI data suggesting the Asn463 glycan does not hinder VRC01_GL_ binding in the context of the 426c core and that this site is glycosylated.

### VRC01_GL_ Fab bound to the Asn276 glycan-containing 426c core construct

A hallmark of VRC01-class bnAb maturation is the shortening of the CDRL1 loop length and/or the addition of glycine residues, both of which have been proposed to enable accommodation of the Asn276 glycan near the CD4_BS _(Jardine et al., 2016a; Scharf et al., 2016; Wu et al., 2015; Zhou et al., 2010). Although VRC01_MAT_ was shown to bind to the trimeric Env CD4_BS_ in the presence of glycan Asn276, its removal significantly increased binding affinity and neutralization potency (Jardine et al., 2013; McGuire et al., 2016; McGuire et al., 2013; Medina-Ramírez et al., 2017a; Stamatatos et al., 2017). Removal of the Asn276 NLGS from certain trimeric SOSIP constructs by either N276D or (S/T)278(A/R) mutations significantly enhanced the antigenicity of the VRC01 epitope (McGuire et al., 2016; McGuire et al., 2013). However, when such glycan-depleted trimeric Env constructs were used as immunogens, the antibodies they elicited failed to overcome the glycan present at position Asn276 of wild-type viruses (Briney et al., 2016; Dosenovic et al., 2015). Removal of glycan Asn276 through N276A substitution abrogated VRC01_GL_ interactions, indicating this amino acid residue was critical for binding (McGuire et al., 2016). These observations suggest that initial engagement of VRC01-class bnAb precursors in infected individuals occurs with an asparagine at position 276 and may also be possible, at low levels, in the presence of a glycan at this NLGS (Scharf et al., 2016).

**Figure 3.**
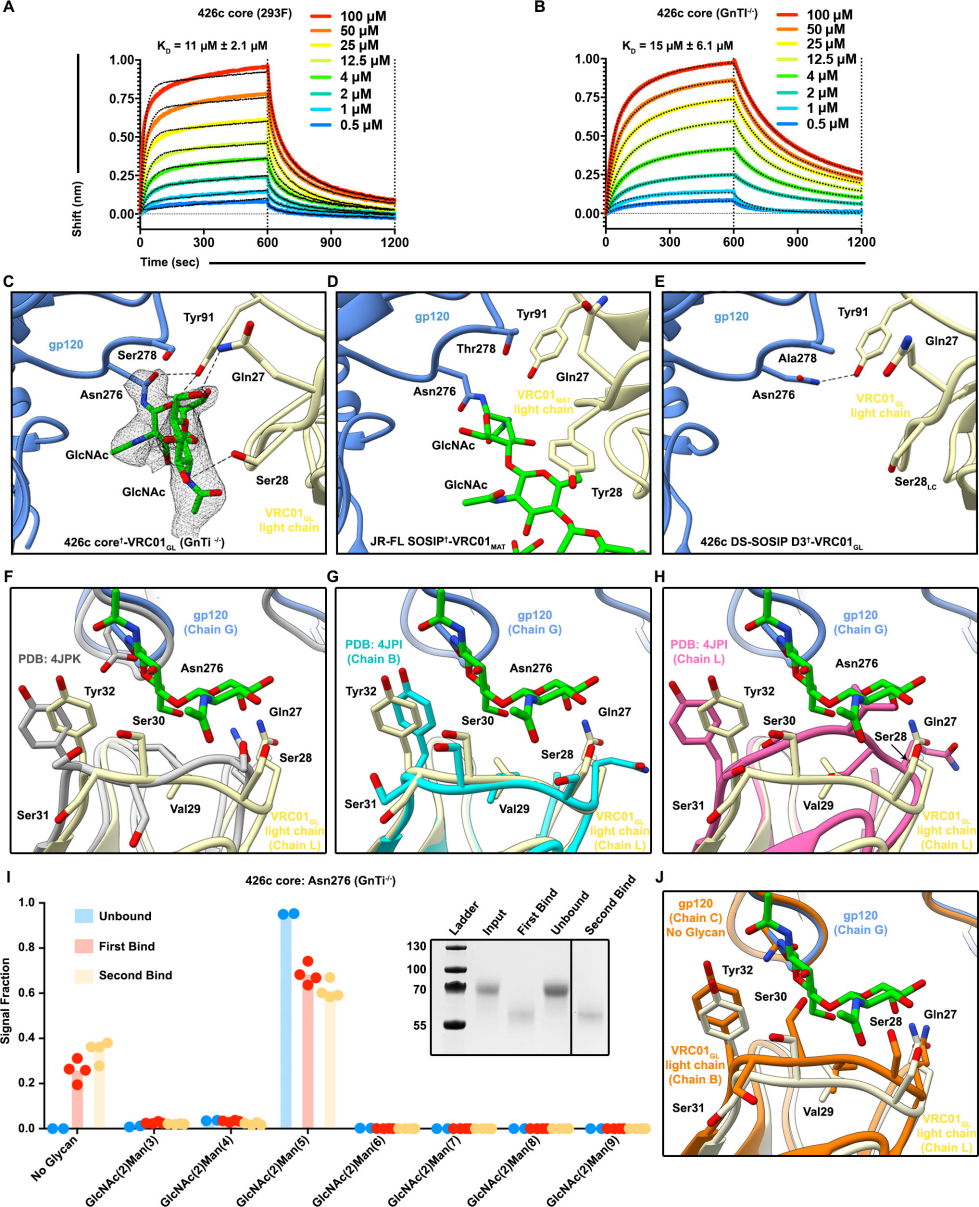
The VRC01_GL_ Fab bound to the 426c core in presence of a glycan at position Asn276. (**A–B**) BLI binding data of the immobilized VRC01_GL_ Fab with the 426c core expressed in either HEK293F (**A**) or HEK293 GnTI^-/-^ cells (**B**). (**C**) Crystal structure of 426c core^†^-VRC01_GL_ highlighting glycan electron density at position Asn276 (grey mesh: 2F_O_-Fc map contoured at 1.0σ) and amino-acid contacts for one molecule of the asymmetric unit. (**D**) Structure of VRC01_MAT_ in complex (crosslinked) with the HIV-1 JR-FL SOSIP trimer (PDB ID: 5FYK) (Stewart-Jones et al., 2016) in the same orientation as in panel (**C**) and focusing on the glycan at position Asn276. (**E**) CryoEM structure of the 426c DS-SOSIP D3^†^-VRC01_GL_ complex in the same orientation as in panel (**C**) and focusing on Asn276. Hydrogen bonds spanning 2.8–3.5 Å are depicted as dashed lines. (**F–H**) Comparison of VRC01_GL_ CDRL1 conformations in the presence or absence of a glycan at position Asn276. In the three panels, gp120 is shown in blue cartoon representation and VRC01_GL_ light chain in light yellow for our crystal structure of 426c core^†^-VRC01_GL_. Residues Gln27 to Tyr32 of VRC01_GL_ light chain are shown as sticks and labeled. (**F**) VRC01_GL_ bound to eODGT6 (PDB ID: 4JPK)(Jardine et al., 2013) is shown in grey. (**G**) Chain B of unliganded VRC01_GL_ (PDB ID: 4JPI) (Jardine et al., 2013) is shown in cyan. (**H**) Chain L of unliganded VRC01_GL_ (PDB ID: 4JPI) (Jardine et al., 2013) is shown in pink. (**I**) Semi-quantitative LC-MS/MS analysis depicting the relative signal intensities for identified Asn276 glycoforms in unbound (blue), after the first binding event (red), and after the second binding event (yellow) fractions taken from VRC01_GL_-based IP experiments. The ‘unbound’ material indicates 426c core glycoforms that did not bind VRC01_GL_ following three binding events. Colored dots on corresponding histogram bars represent individual values extracted from each experimental replicate, with the bar itself representing the experimental mean signal fraction. (*Inset)* SDS-PAGE depicting the average molecular weight difference between wild-type 426c core species in ‘unbound’ flow-through and ‘bound’ elution fractions. (**J**) Structural comparison of VRC01_GL_ CDRL1 conformations in the presence or absence of a glycan at position Asn276 in each of the molecules present in the asymmetric unit of our 426c core^†^-VRC01_GL_ crystal structure.

Considering the reduced glycan shielding of the 426c strain, the deletion of variable loops 1, 2, and 3 in our 426c core constructs, and the minimal impact V5 loop NLGSs had on VRC01_GL_ binding, we tested whether the wild type 426c core construct could interact with VRC01_GL_ in the presence of the Asn276 NLGS (Figure 1—figure supplement 1). BLI analysis revealed VRC01_GL_ bound similarly to both the HEK293F and HEK293 GnTi^-/-^-expressed 426c cores with equilibrium dissociation constants of 11 μM and 15 μM, respectively (Figure 3A,B). The crystal structure of the disulfide engineered 426c core^†^-VRC01_GL_ complex expressed in GnTi^-/-^ cells further reveals the presence of a resolved glycan at position Asn276 in one of the two molecules of the asymmetric unit, indicating VRC01_GL_ bound to both Asn276 glycosylated and unglycosylated species (Figure 3C–J).

**Figure 4.**
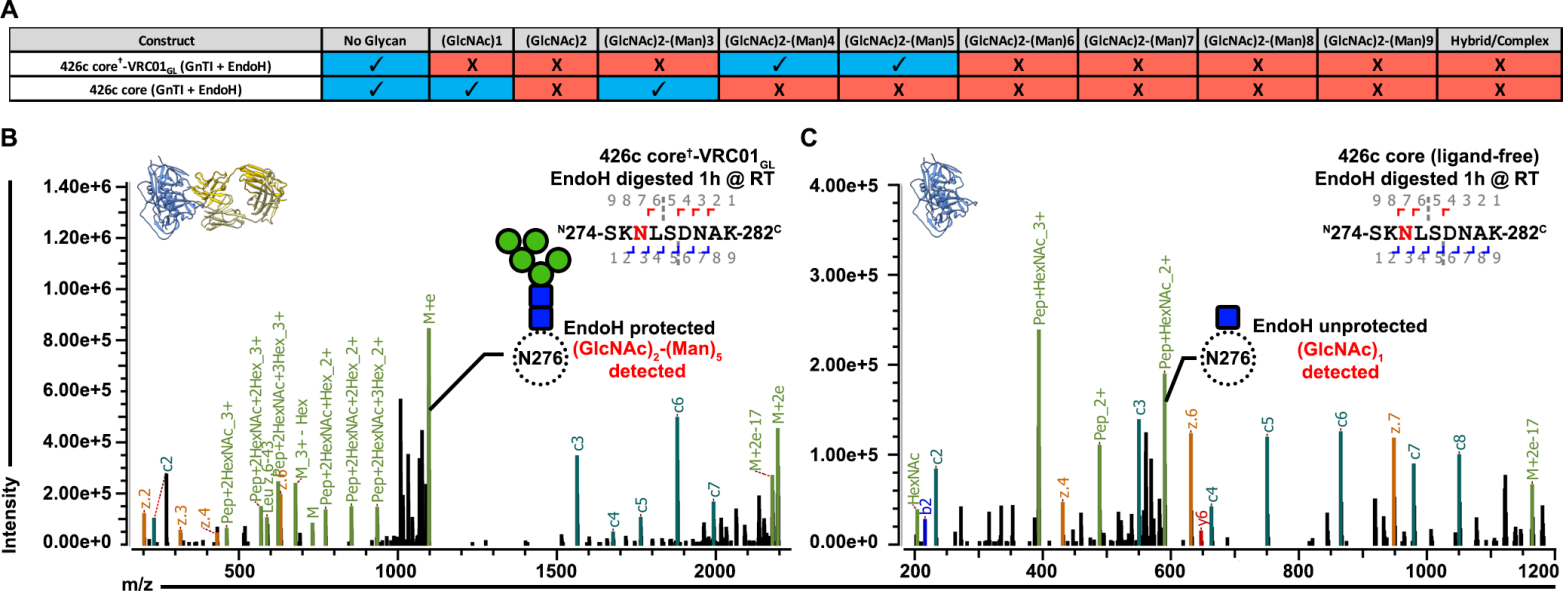
VRC01_GL_ binding in the presence of a glycan at position Asn276 and protection against EndoH-mediated digestion. (**A**) Summary of identifications for 426c Asn276 glycopeptides. 426c core constructs that were subjected to qualitative LC-MS/MS are indicated on the left. Glycopeptide identifications detected using the Byonic software (Bern et al., 2012) are listed in blue and denoted with a check-mark (✓). (**B–C**) Representative LC-MS/MS spectra from panel (**A**) of glycan Asn276 identifications from the cross-linked 426c core^†^-VRC01_GL_ and unliganded 426c core complex following EndoH digestion. The top-left ribbon diagram corresponds to the sample analyzed. The LC-MS/MS fragmentation pattern is indicated in the top-right inset. A graphical depiction of the Asn276 residue (dotted circle) and its associated identified glycan (blue: N-Acetylglucosamine, green : Mannose) are represented on the spectrum. The black line indicates identification of the precursor mass with neutral losses corresponding to the identified glycopeptide. Green peak labels correspond to precursor peptides with/without LC-MS/MS fragmentation occurring within the glycan. Red/orange peak labels represent identified x, y, and z fragments. Blue/teal peak labels highlight identified a, b, and c fragments. After EndoH digestion, a (GlcNAc)_2_-(Man)_5_ glycan was the predominant glycoform identified at position Asn276 with the sample used for crystallization (**B**) whereas a (GlcNAc)_1_ glycan prevailed with the unliganded 426c core (**C**).

Analysis of this structure highlights distinct sets of interactions observed between the light chains of VRC01_GL_ or VRC01_MAT_ and glycan Asn276, which is rotated ~90˚ when comparing the two structures (Figure 3C–D). In line with our previous observation that Asn276 is important for VRC01_GL_ recognition (McGuire et al., 2016), we observed that Asn276 is hydrogen bonded to the VRC01_GL_ light chain residue Tyr91 in our 426c core^†^-VRC01_GL_ and 426c DS-SOSIP D3^†^-VRC01_GL_ structures, but not in the JR-FL SOSIP-VRC01_MAT_ crosslinked complex structure (Figure 3C–E) (PDB: 5FYK) (Stewart-Jones et al., 2016). The CDRL1 of VRC01_GL_ has been shown to adopt multiple conformations both when unliganded and when bound to eOD-GT6; the latter of which lacked a glycan at position Asn276 (Jardine et al., 2013). The CDRL1 loop of NIH45-46_GL_ (a germline VRC01-class antibody) bound to the 426c core TM4 adopts a similar orientation as the one observed for the CDRL1 loop of VRC01_GL_ bound to eOD-GT6 (Scharf et al., 2016) (Jardine et al., 2013) (Supplementary file 2). Comparisons between the structures of our 426c core^†^-VRC01_GL_ complex, a putatively authentic germline VRC01/eOD-GT6 complex (PDB ID 4JPK [Jardine et al., 2013]) and the unliganded VRC01_GL_ (PDB ID 4JPI) (Jardine et al., 2013), indicate that the CDRL1 of VRC01_GL_ accommodates the Asn276 oligosaccharide in a conformation similar to the CDRL1 of unliganded VRC01_GL_ (chain B) (Supplementary file 2)(Jardine et al., 2013), but differs from a complex with a gp120 core lacking this glycan (Figure 3F–H). This observation supports disulfide crosslinking of 426c core^†^-VRC01_GL_ did not distort the binding interface into a non-native conformation for the accommodation of glycan Asn276 in our structure (Figure 3C). LC-MS/MS analysis of the sample used for crystallization revealed *N*-linked carbohydrates at position Asn276 of 426c core^†^-VRC01_GL_ ranged from (GlcNAc)_2_-(Man)_4_ to (GlcNAc)_2_-(Man)_5 _(Figure 4A,B, Figure 2—figure supplement 1E). Unglycosylated Asn276 peptides were also identified, corroborating the presence of two populations of molecules in the crystal structure and the ability of VRC01_GL_ to recognize both species (Figure 4A, Figure 2—figure supplement 1F).

Despite the EndoH treatment used to promote crystallization of 426c core^†^-VRC01_GL_, no (GlcNAc)_1_ glycopeptides were detected at the Asn276 NLGS by LC-MS/MS (Figure 3A). In contrast, we detected digested glycopeptides containing (GlcNAc)_1_ moieties for other NLGSs, confirming the efficiency of the EndoH treatment (Figure 2—figure supplement 1B). These observations validated the Asn276 glycan density observed in the crystal structure and suggested that bound VRC01_GL_ protected the glycan Asn276 from enzymatic digestion (Figure 4A,B), but not other oligosaccharides, such as glycan Asn463 (Figure 2E,G and Figure 2—figure supplement 1B). We further corroborated this hypothesis by analyzing EndoH-treated 426c core in the absence of co-expressed VRC01_GL_ and confirmed the presence of (GlcNAc)_1_ moieties at position Asn276 by LC-MS/MS (Figure 3A,C), supporting that digestion of this glycan was possible if not sterically hindered by the binding of this Fab (Yet et al., 1988).

To probe whether the disulfide cross-link promoted artificial accommodation of glycan Asn276, we performed an additional analysis with samples obtained from IP experiments using the 426c core construct lacking the G459C mutation. 426c core samples from the ‘unbound’ flow-through and ‘bound’ elution fractions had distinct migration profiles by SDS-PAGE (Figure 3I), with the bound fraction exhibiting higher electrophoretic mobility than the unbound species. LC-MS/MS revealed the bound fraction was enriched for unglycosylated Asn276 peptides, suggesting this subspecies was the preferred VRC01_GL_ binder (Figure 3I). This result corroborates reports of VRC01_GL_ binding occurring preferentially in the absence of a glycan at position 276 (Jardine et al., 2013; McGuire et al., 2016; McGuire et al., 2013; Medina-Ramírez et al., 2017a; Scharf et al., 2016; Stamatatos et al., 2017). However, we also detected that the majority of the Asn276 peptide signal was of the (GlcNAc)_2_-(Man)_5_ glycoform in bound fractions (approximately twice as much as the unglycosylated Asn276 signal) (Figure 3I), suggesting VRC01_GL_ could indeed bind in the presence of this glycan and in the absence of an engineered cross-link. This experiment, along with our crosslinked 426c core^†^-VRC01_GL_ crystal structure and qualitative LC-MS/MS, confirm both the glycosylated (Figure 3C,F,G,H,I) and unglycosylated Asn276 glycoforms are present following expression and that VRC01_GL_ could accommodate both. In summary, VRC01_GL_ bound a 426c core with wild-type NLGSs, was sterically compatible with glycans present at positions Asn276 and Asn463, and strictly interacted with a subspecies of gp120 lacking a glycan at position Asn460.

### Modulation of glycan composition altered VRC01_GL_ antibody recognition of the 426c core

Irrespective of the chosen expression system (HEK293F or HEK293 GnTI^-/-^), our LC-MS/MS analyses showed that 426c core constructs all contained detectable levels of both the unglycosylated and the (GlcNAc)_2_-(Man)_5_ oligosaccharide variants at position Asn276 (Figure 4A–B, Figure 3I, Figure 2—figure supplement 1D–G). Since (GlcNAc)_2_-(Man)_5_ is a short glycan produced in mammalian cells (Hossler et al., 2009), and was the major detectable glycosylated form in VRC01_GL_-based IP ‘bound’ elution fractions, we interrogated whether differential expression conditions known to enrich for (GlcNAc)_2_-(Man)_9_ glycans could negatively impact the binding of VRC01_GL_ IgGs to 426c core. We thus compared VRC01_GL_ binding to HEK293 GnTI^-/-^-produced 426c core constructs expressed in the absence or presence of 100 µM kifunensine to yield a range of (GlcNAc)_2_-(Man)_5_ to (GlcNAc)_2_-(Man)_9_ glycans or to enrich for (GlcNAc)_2_-(Man)_9_ glycans, respectively (Depetris et al., 2012).

**Figure 5.**
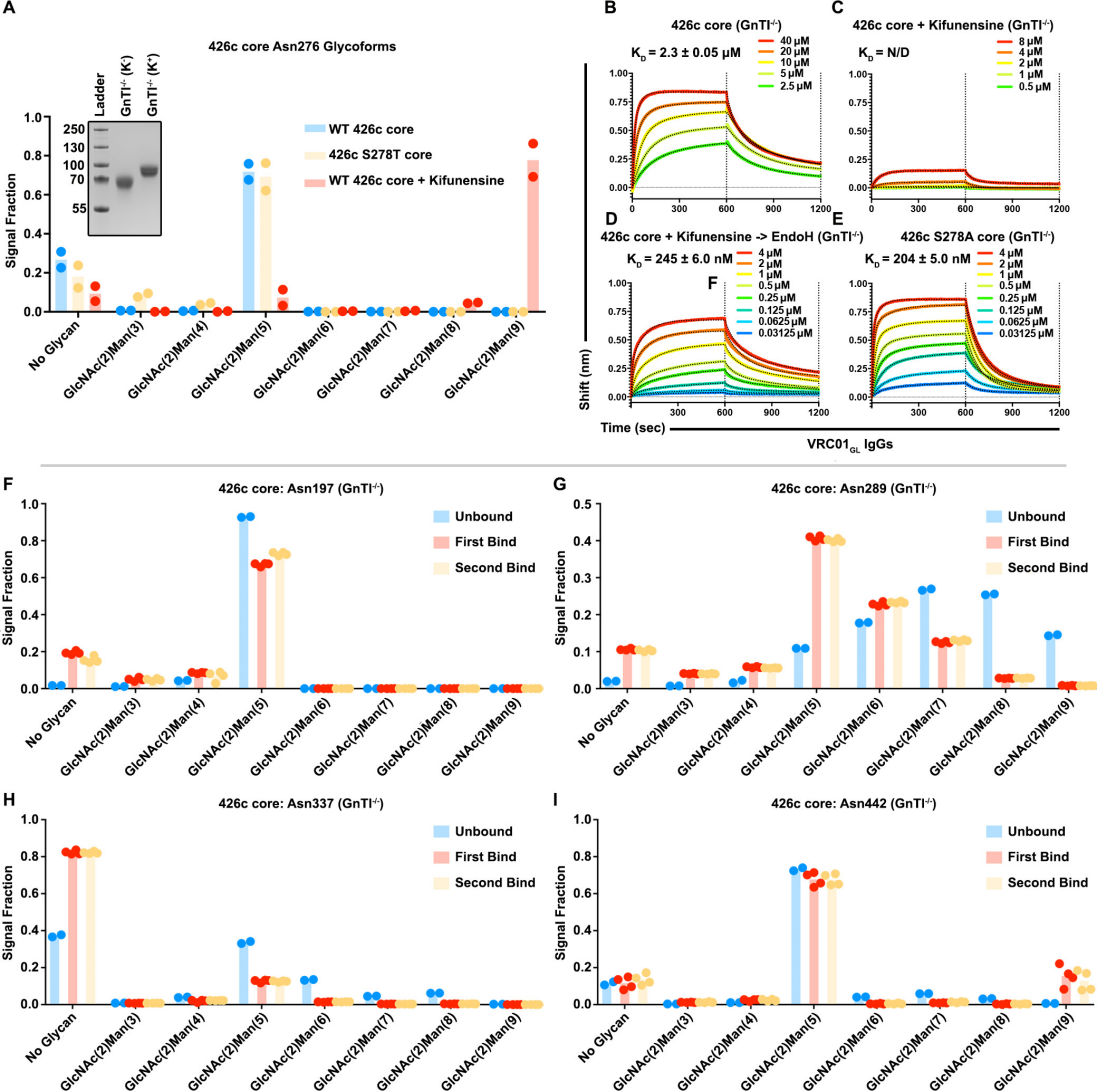
Glycan length impacted VRC01_GL_ IgG recognition of the 426c core. (**A**) Semi-quantitative LC-MS/MS analysis depicting the relative signal intensities for identified Asn276 glycoforms in 426c core (blue), 426c S278T core (yellow), and 426c core expressed in the presence of 100 µM kifunensine (red). (*Inset*) SDS-PAGE demonstrating the molecular weight difference between the 426c core expressed in the absence (K^-^) or presence (K^+^) of 100 µM kifunensine. The molecular weights of the protein standards are indicated on the left. (**B–E**) BLI binding data and determined equilibrium dissociation constant values of VRC01_GL_ IgG binding to the 426c core expressed using HEK293 GnTI^-/-^ cells (**B**), the 426c core expressed using HEK293 GnTI^-/-^ cells in the presence of 100 μM kifunensine (**C**), the 426c core expressed using HEK293 GnTI^-/-^ cells and digested with EndoH (**D**), and the 426c S278A core expressed using HEK293 GnTI^-/-^ cells (**E**). The concentrations of 426c core injected are indicated on each panel. Fitted curves are colored as black dotted lines. The vertical dotted lines indicate the transition between association and dissociation phases. N/D: not determined. (**F–I**) Semi-quantitative LC-MS/MS analysis depicting the relative signal intensities of unbound (blue), first binding event (red), and second binding event (yellow) fractions taken from VRC01_GL_-based IP experiments. Glycoforms were analyzed for NLGSs Asn197 (**G**), Asn289 (**H**), Asn337 (**I**), and Asn442 (**J**). Colored dots in panels A and F-I represent individual values extracted from each experimental replicate, with the bar itself representing the experimental mean signal fraction.

The efficacy of this strategy was confirmed by two orthogonal methods: (1) SDS-PAGE, which demonstrated the two expression conditions yielded samples with distinct migration profiles, and (2) LC-MS/MS, which confirmed enrichment for (GlcNAc)_2_-(Man)_9_ glycans in the presence of kifunensine (Figure 5A, Figure 5—figure supplement 1A–C). Importantly, binding affinities for the 426c core were improved by ~10 fold in the context of immobilized full-length VRC01_GL_ IgGs relative to immobilized VRC01 Fabs. The 426c core expressed using HEK293 GnTI^-/-^ in the absence of kifunensine bound VRC01_GL_ IgG with a K_D_ of 2 µM (Figure 5—figure supplement 1B, Supplementary file 3), whereas binding was significantly reduced when the 426c core was expressed in the presence of kifunensine (Figure 5C, Supplementary file 3). The VRC01_GL_ IgG binding affinity to 426c core expressed in GnTI^-/-^ cells in the presence of kifunensine was enhanced following treatment with EndoH (K_D_ = 245 nM, Figure 5D, Supplementary file 3), confirming recognition of gp120 by VRC01_GL_ IgGs can occur in the presence of a proximal GlcNAc at position Asn276. This VRC01_GL_ IgG binding affinity was similar to the HEK293 GnTI^-/-^-expressed 426c S278A core construct (K_D_ = 204 nM), although the kinetics of binding differed for the two samples (Figure 5E).

**Figure 5—figure supplement 1.**
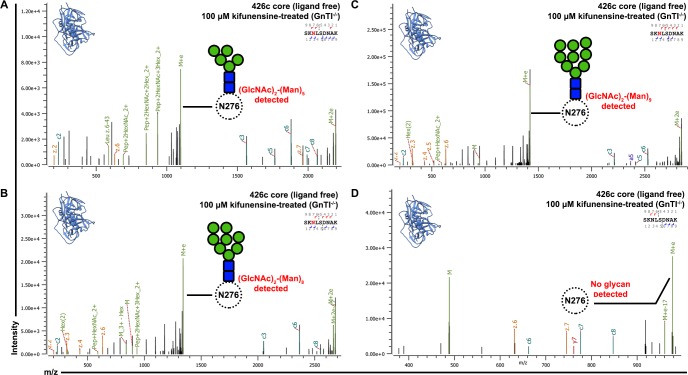
LC-MS/MS glycan identifications of kifunensine-treated 426c core constructs. Representative LC-MS/MS spectra of glycan Asn276 identifications from the kifunensine-treated 426c core sample used for BLI experiments. The top-left ribbon diagram corresponds to the sample analyzed. The LC-MS/MS fragmentation pattern is indicated in the top-right inset. A graphical depiction of the Asn276 residue (dotted white circle) and its associated identified glycan (blue: N-acetyl glucosamine; green: mannose;) are represented on the spectrum. Green peak labels correspond to precursor peptides with/without LC-MS/MS fragmentation occurring within the glycan. Red/orange peak labels represent identified x, y, and z fragments. Blue/teal peak labels highlight identified a, b, and c fragments. (**A–D**) Representative LC-MS/MS spectra of an Asn276 glycopeptide with a (GlcNAc)_2_-(Man)_5_ oligosaccharide from the 426c core expressed in GnTI^-/-^ cells in the presence of 100 µM kifunensine (**A**), an Asn276 glycopeptide with a (GlcNAc)_2_-(Man)_8_ oligosaccharide (**B**), an Asn276 glycopeptide with a (GlcNAc)_2_-(Man)_9_ oligosaccharide (**C**), and an unglycosylated Asn276 peptide (**D**). All these identifications were made from the same kifunensine-treated sample.

**Figure 5—figure supplement 2.**
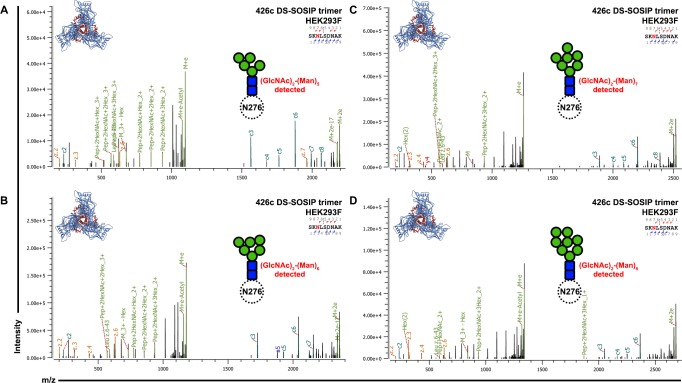
Trimeric 426c DS-SOSIP has a variable glycan length at position Asn276. Representative LC-MS/MS spectra of glycan Asn276 identifications from the 426c DS-SOSIP. The top-left ribbon diagram corresponds to the sample analyzed. The LC-MS/MS fragmentation pattern is indicated in the top-right inset. A graphical depiction of the Asn276 residue (dotted white circle) and its associated identified glycan (green circle = Mannose; blue = N Acetylglucosamine) are represented on the spectrum. Green peak labels correspond to precursor peptides with/without LC-MS/MS fragmentation occurring within the glycan. Red/orange peak labels represent identified x, y, and z fragments. Blue/teal peak labels highlight identified a, b, and c fragments. (**A–D**) Representative LC-MS/MS spectrum of an Asn276 glyco-peptide of a detectable (GlcNAc)_2_-(Man)_5_ glycan (**A**), a (GlcNAc)_2_-(Man)_6_ glycan (**B**), a (GlcNAc)_2_-(Man)_7_ glycan (**C**), and a (GlcNAc)_2_-(Man)_8_ glycan (**D**).

Previous studies demonstrated enhanced VRC01_MAT_ binding following glycan Asn276 removal, although VRC01_MAT_ could also accommodate an Asn276 glycan when present (Jardine et al., 2013; McGuire et al., 2013; Medina-Ramírez et al., 2017a); McGuire et al., 2016; Scharf et al., 2016; Stamatatos et al., 2017; Zhou et al., 2017). Similarly to VRC01_MAT_, VRC01_GL_ binding was previously detected in the absence of the Asn276 glycan (McGuire et al., 2016; McGuire et al., 2013). In this present study, VRC01_GL_ binding was also observed under typical expression conditions with the native Asn276 NLGS retained, but was reduced following expression in the presence of kifunensine (Supplementary file 3, Figure 5A,B,C, Figure 5—figure supplement 1A,B,E,F). This may indicate that kifunensine treatment results in more efficient glycosylation of some NLGSs or that differences in glycan length influence recognition of VRC01_GL_-class antibodies to the CD4_BS_, or both (Figure 5A). In line with these hypotheses, the VRC01_GL_*-*based IP and subsequent semi-quantitative LC-MS/MS of the 426c core expressed in the absence of kifunensine revealed a consistent preference for short and/or unglycosylated species bound to VRC01_GL_ (Figure 3I, Figure 5F,G,H,I). These findings explain the observed increase in electrophoretic mobility of 426c core species in VRC01_GL_-based IP fractions that bound this antibody compared to the unbound fraction (Figure 3I).

While many 426c core glycans are likely to be affected by either kifunensine or EndoH treatment, the Asn276 oligosaccharide is expected to have a pronounced negative effect on VRC01_GL_ binding due to its direct overlap with the VRC01_GL_ epitope (Stewart-Jones et al., 2016; Zhou et al., 2017). The 426c core^†^-VRC01_GL_ crystal structure does not resolve ordered mannose rings at position Asn276, despite their high detected abundance by LC-MS/MS, and thus only the two proximal GlcNAc moieties were modeled in our structure (Figure 4C). This indicates the Asn276 mannose moieties are likely not directly involved in binding to VRC01_GL_ light chain, but rather act as a steric barrier VRC01_GL_ must overcome to interact with the CD4_BS_. Qualitative LC-MS/MS analyses of the 426c DS-SOSIP trimer, expressed in the absence of kifunensine, revealed longer glycans at the Asn276 NLGS, which correlated with a poorer binding affinity, relative to the 426c core also expressed in the absence of kifunensine (Figure 1A, Figure 5—figure supplement 2). Indeed, interactions with the Asn276-linked mannose moieties might be restricted to VRC01_MAT_, as these rings are well-resolved in corresponding crystal structures. VRC01_GL_-specificity and compatibility for proximal GlcNAcs is made evident by the increased affinity of VRC01_GL_ for the 426c core following digestion with EndoH (Figure 5D). These results indicate binding of several germline antibodies to gp120 core constructs could potentially be modulated by tailoring protein expression conditions, oligosaccharide length, and/or by endoglycosidase treatment, as opposed to strict mutations aimed at abolishing NLGSs (Kong et al., 2010).

### The amino acid sequence of an intact 426c core Asn276 NLGS modulated VRC01_GL_ antibody recognition

**Figure 6.**
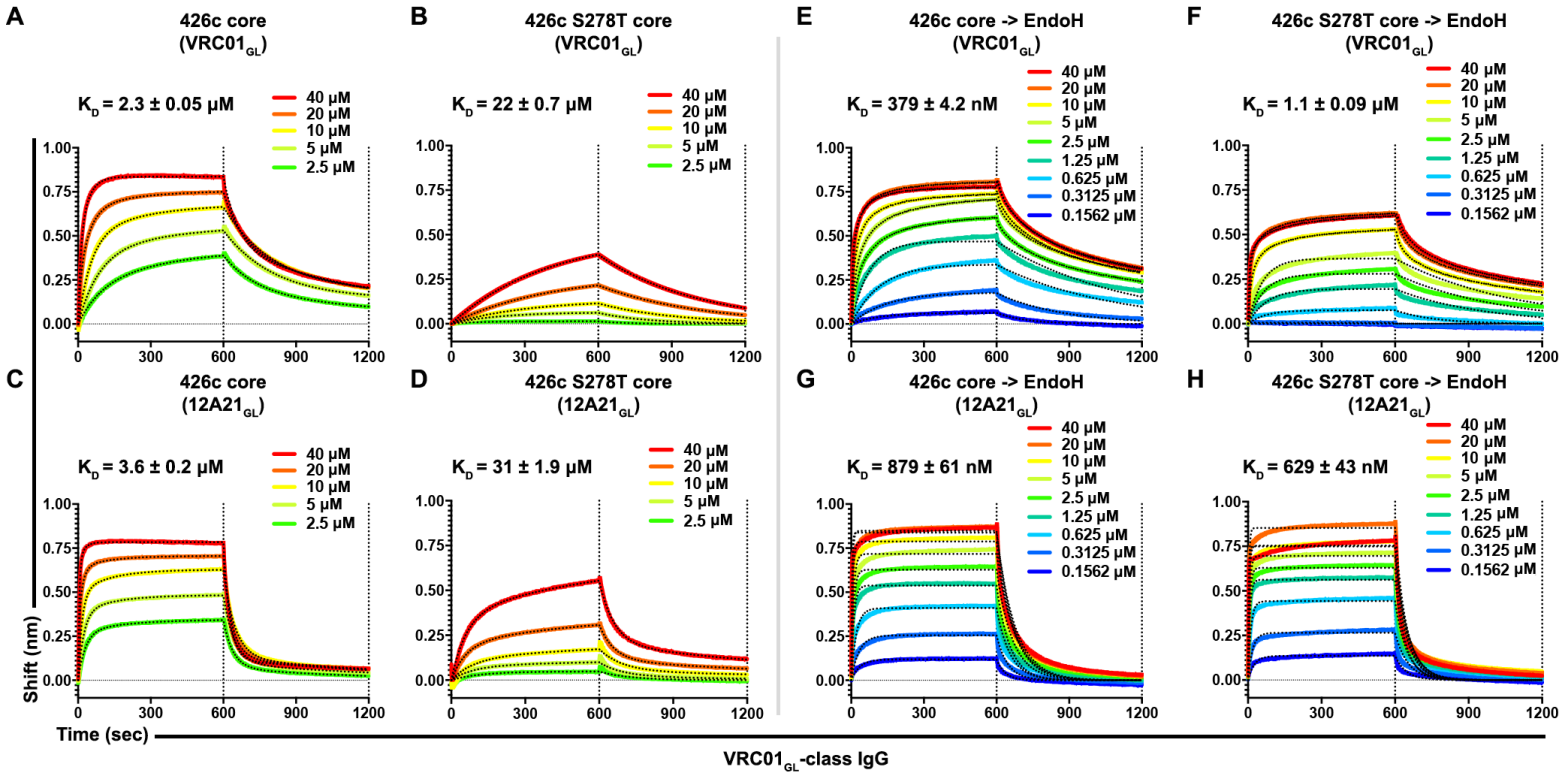
Asn276 glycosylation frequency modulated VRC01_GL_-class IgGs recognition of the 426c core. (**A–D**) BLI binding data and associated K_D_ values of 426c core constructs, expressed in HEK293 GnTI^-/-^ cells, with two immobilized VRC01_GL_-class IgGs. VRC01_GL_ IgG binding was assessed against the 426c core (**A**) and the 426c S278T core (**B**). 12A21_GL_ binding to the 426c core (**C**) and 426c S278T core (**D**) were also tested. (**E–H**) BLI binding data and corresponding K_D_ values of 426c core constructs, expressed using HEK293 GnTI^-/-^ cells and treated with EndoH, with VRC01_GL_-class IgGs. VRC01_GL_ IgG binding was assessed against the EndoH-treated 426c core (**E**) and the 426c S278T core (**F**). 12A21_GL_ interactions with the EndoH-treated 426c core (**G**) and 426c S278T core (**H**) were also tested. The concentrations of 426c core injected and the color key are indicated on each panel. Fit curves are colored as black dotted lines. The vertical dotted lines indicate the transition between association and dissociation phases.

Considering our prior work demonstrating that amino acid composition of the Asn276 NLGS impacted VRC01_GL_ recognition independently of glycan presence (McGuire et al., 2016), and our observation that VRC01_GL_ could bind to 426c core in the presence of a native NLGS at position Asn276 (with a preferential selection for the unglycosylated variant), we decided to test whether altering the identity of the Asn276 NLGS at the 278 position could impact VRC01_GL_-class antibody binding. Whereas 82% of sequenced HIV-1 clades harbor a threonine residue at position 278 of gp120, 426c gp120 contains a serine at this position. Since recent reports suggested NXT NLGS sites are more efficiently glycosylated relative to NXS sites (Huang et al., 2017), we compared the binding kinetics of full-length VRC01_GL_-class IgGs, VRC01_GL_ and 12A21_GL_, to HEK293 GnTI^-/-^-expressed 426c core and an 426c S278T core mutant (Figure 1—figure supplement 1 and Figure 1—figure supplement 2). BLI experiments demonstrated the S278T mutation reduced the equilibrium dissociation constant by 10-fold relative to the wild type NLGS, mainly by decreasing association kinetics (Figure 6A–D, Figure 5—figure supplement 2A). The S278T mutation did not abrogate VRC01_GL_-class antibody binding, despite an expected improvement in Asn276 glycosylation efficiency (Huang et al., 2017). Moreover, semi-quantitative LC-MS/MS revealed (GlcNAc)_2_-(Man)_5_ oligosaccharides were predominantly detected at position Asn276 in the S278T mutant, though some unglycosylated peptides were still present at low levels (Figure 5A, Figure 2—figure supplement 1H,I).

**Figure 6—figure supplement 1.**
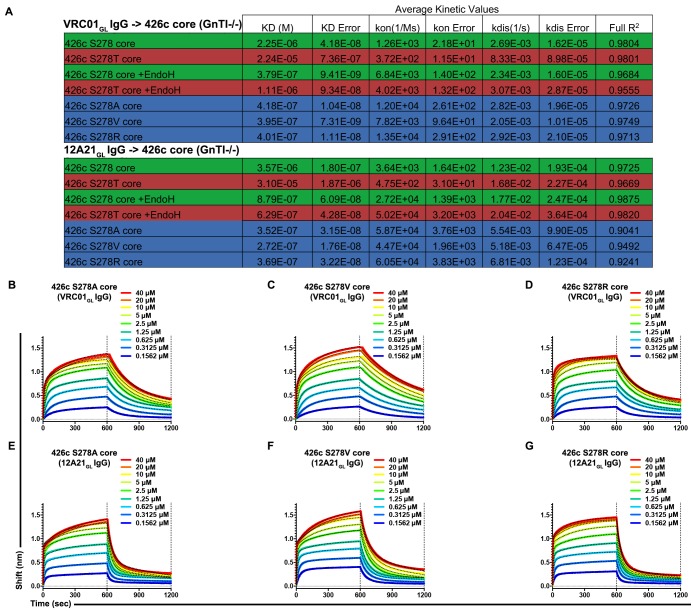
VRC01_GL_ binding affinity was not significantly affected by the identity of the residue 278 in the absence of a glycan at position Asn276. (**A**) BLI kinetics parameters determined from panels (**B–G**) and Figure 6. (**B–D**) BLI binding data and determined equilibrium dissociation constants for VRC01_GL_ IgG binding to 426c S278A core (**A**), 426c S278V core (**B**), and 426c S278R core. (**E–G**) BLI binding data and determined equilibrium dissociation constants of 12A21_GL_ IgG binding to 426c S278A core (**A**), 426c S278V core (**B**), and 426c S278R core. The concentrations of 426c core injected are indicated on each panel. Fit curves are colored black. The vertical dashed lines indicate the transition between association and dissociation phases.

To rule out any potential impact residue identity at position 278 might have on VRC01_GL_-class antibody binding affinities (irrespective of glycosylation status), we mutated the Asn276 NLGS using substitutions S278A, S278V, and S278R (Figure 1—figure supplement 1). Although the magnitude of binding was improved relative to both 426c core constructs containing a glycan at position Asn276, no appreciable difference in affinities was detected among any of these NLGS-depleted 426c core mutants (Figure 6—figure supplement 1A–F). Since binding was reduced following introduction of the S278T mutation, these results collectively suggest that a significant fraction of the interactions observed by BLI between the 426c core (containing an intact Asn276 NXS NLGS) and VRC01_GL_ occurred with the unglycosylated Asn276 subspecies. This construct is expected to be less-frequently glycosylated at position Asn276 relative to the S278T mutant (Huang et al., 2017). However, VRC01_GL_-class IgG binding was also detected with the 426c S278T core (Figure 6A–D, Figure 6—figure supplement 1A), and EndoH treatment (which retains core GlcNAcs) of both the S278 and S278T constructs significantly improved IgG binding relative to their untreated counterparts (Figure 6E–H, Figure 6—figure supplement 1A). Although these findings support that VRC01_GL_-class binding is dampened by the Asn276 mannose moieties , interactions were still possible, as underscored by the presence of this carbohydrate in the crystal structure (Figure 3C), the EndoH protection assay (Figure 4B,C), and VRC01_GL_-based IP LC-MS/MS analyses of non-crosslinked samples (Figure 3I). The ability of VRC01_GL_-class antibodies to recognize a CD4_BS_ containing an NLGS at position Asn276 and the linked oligosaccharide is unprecedented, and highlights a potentially unique feature of the 426c core construct containing all native NLGSs for engagement of VRC01_GL_ antibodies.

## Discussion

Broad-spectrum and potent neutralization of HIV-1 by naturally occurring VRC01 bnAbs targeting the CD4_BS_ is possible in humans (Huang et al., 2016). However, mature VRC01-class bnAbs are produced only in a small fraction of infected individuals and only after up to a decade following the initial infection (Wu et al., 2015). Due to the negligible binding of germline VRC01-class antibodies to ‘wild-type’ stabilized prefusion-closed SOSIP trimers, we sought out to understand putative mechanisms of primary engagement of this class of germline antibodies by various HIV-1 immunogens. To this end, we first engineered a disulfide bond between the glycan depleted 426c DS-SOSIP D3 and VRC01_GL_ to promote complex formation. This tethering approach was recently described for VRC01_MAT_ and led to native structures that do not suffer from any distortions (Stewart-Jones et al., 2016). It is unlikely that the engineered disulfide would force the VRC01_GL_ and 426c gp120 to interact in a homogeneous way. Indeed, an engineered disulfide bond would only prevent dissociation of the two proteins (similarly to what we previously described for mature VRC01 [Stewart-Jones et al., 2016]), but will not force them to interact uniformly (Stewart-Jones et al., 2016). Using this crosslinking strategy, we demonstrated that VRC01_GL_ bound to the deglycosylated 426c DS-SOSIP D3^†^ trimer in the absence of a formed bridging sheet and that this binding stabilized surrounding CD4_BS_ carbohydrates at position Asn197 and Asn386. Considering there was a minor enrichment for the unglycosylated Asn197 glycoform in ‘bound’ fractions following VRC01_GL_-based immunoprecipitation of the 426c core, there could be an entropic cost associated with binding in the presence of this particular oligosaccharide. Additionally, we found that 426c naturally lacks glycans at positions Asn234 and Asn362, which likely enhanced accessibility of the CD4_BS_ to bnAbs and could affect processing of nearby carbohydrates (Behrens et al., 2018), including glycan Asn276. We propose this is one of the reasons explaining the ability of 426c DS-SOSIP D3 and 426c core constructs to bind VRC01_GL_-class antibodies.

Immunogen design efforts focusing on VRC01-class bnAbs have thus far largely relied on the mutagenic removal of NLGSs during priming followed by their reinsertion during subsequent boosts (McGuire et al., 2013; Medina-Ramírez et al., 2017a; Zhou et al., 2017). This strategy may not recapitulate the conditions of *bona fide* infections, as several CD4_BS_ NLGSs are conserved amongst circulating HIV-1 strains (Crooks et al., 2015; Lavine et al., 2012; Pritchard et al., 2015; Stewart-Jones et al., 2016). The complete absence of these NLGSs during the priming phase may also remove a selection pressure guiding proper accommodation of these glycans during antibody affinity maturation, and could explain the limited success achieved thus far to elicit VRC01-class bnAbs capable of neutralizing natively glycosylated wild-type viruses. As a result, alternative priming and boost strategies retaining these native NLGSs may need to be considered.

Specifically, HIV-1 gp120 core constructs have previously been shown to have differential processing of NLGSs around the CD4_BS_ relative to their trimeric SOSIP counterparts (Bonomelli et al., 2011). We observed that reintroduction of 426c gp120 V5 loop NLGSs did not negatively impact VRC01_GL_ binding to the 426c core in contrast with what was detected for trimeric 426c gp140 (McGuire et al., 2013). We found that VRC01_GL_ Fabs and IgGs could bind to the 426c core (containing a wild-type Asn276 NLGS) but, as expected, not to a trimeric 426c DS-SOSIP construct containing all wild-type NLGSs (Huang et al., 2016). We put forward this interaction is likely permitted in the 426c core in part due to the absence of variable loops 1, 2, and 3, and could potentially be further augmented by the natural absence of glycans at position Asn234 and Asn362. It is also possible that stabilized trimeric pre-fusion closed SOSIP constructs impart additional negative steric effects not present in the monomeric gp120 core. This is supported by our observation that there is a reduced amount of trimming of the Asn276 glycan in the 426c DS-SOSIP trimer relative to the 426c core monomer. This could arise from steric properties of the trimer which would be absent in soluble monomeric constructs, thereby limiting unfettered access of the glycosylation machinery to this site during expression. It also remains to be determined if the conformation of the gp120 bridging sheet influences the recognition efficacy of this germline antibody.

We found that VRC01_GL_ binding to 426c core constructs preferentially occurred using expression systems allowing for production of short glycans and provided a structural framework for accommodation of the Asn276 oligosaccharide by the VRC01_GL_ CDRL1. As is the case for VRC01_MAT_ (Jardine et al., 2013; McGuire et al., 2016; McGuire et al., 2013; Medina-Ramírez et al., 2017a; Stamatatos et al., 2017), we would like to emphasize that the unglycosylated Asn276 variant is the preferred VRC01_GL_ binding partner and likely had an important contribution to the interactions observed by BLI. However, we detected glycosylation at position Asn276, both structurally and by LC-MS/MS following VRC01_GL_-based IP experiments, in the VRC01_GL_-bound fractions, in the presence and absence of cysteine cross-linking, respectively. This indicated accommodation of this oligosaccharide could occur during VRC01_GL_ binding in the absence of CDRL1 loop shortening and/or glycine addition, arguing in favor of its retention in epitope-based constructs derived from the 426c strain of HIV-1.

The structural and biophysical data reported here provide a foundation for understanding how bnAbs targeting the HIV-1 CD4_BS_ may be elicited in humans in the presence of a native Asn276 NLGS. We demonstrated structurally that VRC01_GL_ binding to a 426c core is possible and appears to occur both in the absence and presence of a glycan at position Asn276, supporting recently proposed hypotheses (Jardine et al., 2016b; Scharf et al., 2016). Introduction of an NXS NLGS at position Asn276 of g120 core constructs lacking selected variable loops and in absence of glycans Asn234, Asn362(363), and Asn460 could prove a useful strategy to elicit VRC01-class antibodies during the priming phase of immunization. Additionally, the stark differences in binding observed between VRC01_GL_ and 426c DS-SOSIP or 426c core constructs suggests ‘germline-targeting’ vaccine design strategies starting with a gp120 immunogen may be a promising alternative to current priming strategies focusing on glycan-depleted HIV-1 SOSIP trimers. Indeed, a remarkable success using site-directed epitope-based immunogens targeting other antigenic regions of HIV-1 Env has been recently achieved (Xu et al., 2018). In the context of VRC01_GL_-targeted immunogens, the use of insect cell expression systems may further benefit recognition of the HIV-1 CD4_BS_ due to the abundance of paucimannose sugars (Altmann et al., 1999). This approach has already been reported to enhance the immunogenicity of the CD4_BS_ for other HIV-1 antibodies, but has yet to be specifically shown for antibodies of the VRC01 lineage (Kong et al., 2010). We highlight here the advantages of using an HIV-1 426c core construct for enhancing VRC01-class germline antibody binding relative to the glycan-depleted 426c trimeric Env SOSIP construct and propose that these observations be considered in future HIV-1 vaccine design efforts.

## Materials and methods

**Key resources table:** 

Reagent type (species) or Resource	Designation	Source or reference	Identifiers	Additional information
Software, algorithm	Leginon	doi: 10.1016/ j.jsb.2005.03.010		http://emg.nysbc.org/redmine/projects/leginon/wiki/Leginon_Homepage
Software, algorithm	RELION-2	doi: 10.1016/ j.jsb.2012.09.006	RRID:SCR_016274	http://www2.mrc-lmb.cam.ac.uk/relion/index.php/Main_Page
Software, algorithm	MotionCor2	doi:10.1038/ nmeth.4193		http://emg.nysbc.org/redmine/projects/appion/wiki/Appion_Home
Software, algorithm	GCTF	doi: 10.1016/ j.jsb.2015.11.003	RRID:SCR_016500	https://www.mrc-lmb.cam.ac.uk/kzhang/
Software, algorithm	CTFFIND4	doi:10.1016/ j.jsb.2015.08.008		http://grigoriefflab.janelia.org/ctffind4
Software, algorithm	Frealign	doi: 10.1016/ bs.mie.2016.04.013		http://grigoriefflab.janelia.org/frealign
Software, algorithm	Appion Package	doi: 10.1016/ j.jsb.2009.01.002		http://emg.nysbc.org/redmine/projects/appion/wiki/Appion_Home
Software, algorithm	DoG Picker	doi:10.1016/ j.jsb.2009.01.004		http://emg.nysbc.org/redmine/projects/appion/wiki/Appion_Home
Software, algorithm	Coot	doi: 10.1107/ S0907444910007493	RRID:SCR_014222	http://www2.mrc-lmb.cam.ac.uk/Personal/pemsley/coot/devel/build-info.html
Software, algorithm	Rosetta	doi: 10.1146/annurev. biochem.77.062906 .171838	RRID:SCR_015701	https://www.rosettacommons.org/software
Software, algorithm	UCSF Chimera	doi: 10.1002/jcc.20084	RRID:SCR_004097	http://plato.cgl.ucsf.edu/chimera/
Software, algorithm	PMI-Byonic	doi: 10.1002/ 0471250953.bi1320s40		https://www.proteinmetrics.com/products/byonic/
Software, algorithm	Skyline	doi: 10.1093/ bioinformatics/btq054	RRID:SCR_014080	https://skyline.ms/project/home/software/Skyline/begin.view
Software, algorithm	Octet Data Acquisition	Pall ForteBio	CFR 10.0.3.12d	https://www.fortebio.com/octet-software.html
Software, algorithm	Octet Data Analysis	Pall ForteBio	CFR 10.0.3.1	https://www.fortebio.com/octet-software.html
Software, algorithm	Phaser	doi:10.1107/ S0021889807021206		https://www.phenix-online.org/documentation/reference/phaser.html
Software, algorithm	Phenix.refine	doi:10.1107/ S0907444912001308		https://www.phenix-online.org/documentation/reference/refinement.html
Software, algorithm	GraphPad Prism	GraphPad	RRID:SCR_002798	https://www.graphpad.com/scientific-software/prism/
Software, algorithm	Pymol	Delano, 2002		https://pymol.org/2/
Cell Line (*Homo sapiens*)	HEK293S GnTI-/-	ATCC	ATCC: CRL-3022; RRID:CVCL_A785	https://www.atcc.org/Products/All/CRL-3022.aspx
Cell Line (*Homo sapiens*)	HEK293F	ThermoFisher Scientifc	Cat# R79007	https://www.thermofisher.com/order/catalog/product/R79007
Gene (*Homo sapiens*)	gl VRC01 Igk(3-11)	doi: 10.1126/ science.1192819		
Gene (*Homo sapiens*)	gl VRC01 Igg Fab	doi: 10.1038/ ncomms10618		
Gene (*Homo sapiens*)	gl VRC01 Igg Fab A60C/C98S	This paper		
Gene (*Homo sapiens*)	gl VRC01 Igg	10.1126/science.1192819		
Gene (*Homo sapiens*)	gl 12A21 Igk(1-33)	10.1084/jem.20122824		
Gene (*Homo sapiens*)	gl 12A21 Igg Fab	10.1038/ncomms10618		
Gene (*Homo sapiens*)	gl 12A21 Igg	10.1084/jem.20122824		
Strain, strain background (Human Immunodeficiency Virus-1, Strain: 426 c)	WT_426 c_DS-SOSIP	10.1016/j.cell .2016.07.029		
Strain, strain background (Human Immunodeficiency Virus-1, Strain: 426 c)	426 c_G459C _DS-SOSIP_D3	This paper		
Strain, strain background (Human Immunodeficiency Virus-1, Strain: 426 c)	426 c_WT_ gp120c_core	This paper		
Strain, strain background (Human Immunodeficiency Virus-1, Strain: 426 c)	426 c_G459C _gp120c_core	This paper		
Strain, strain background (Human Immunodeficiency Virus-1, Strain: 426 c)	426 c_S278A _gp120c_core	This paper		
Strain, strain background (Human Immunodeficiency Virus-1, Strain: 426 c)	426 c_S278A_T462A _gp120c_core	This paper		
Strain, strain background (Human Immunodeficiency Virus-1, Strain: 426 c)	426 c_S278A_T465A _gp120c_core	This paper		
Strain, strain background (Human Immunodeficiency Virus-1, Strain: 426 c)	426 c_S278A_T462A_ T465A_gp120c_core	This paper		
Strain, strain background (Human Immunodeficiency Virus-1, Strain: 426 c)	426 c_S278T_gp120c	This paper		
Recombinant DNA reagent	pTT3	PMID: 11788735		https://biochimie.umontreal.ca/en/department/professors/yves-durocher/
Recombinant DNA reagent	pVRC8400	NIH		
Chemical compound, drug	Kifunensine	Sigma-Aldrich	CAS Number 109944-15-2	https://www.sigmaaldrich.com/catalog/product/sigma/k1140?lang=en&region=US&gclid=Cj0KCQjwr53OBRCDARIsAL0vKrNtYwTyRzHU65HyVBwdntcP3kGpZ0ElVwYeSK3OcorLn0wf8U1iMQgaAssSEALw_wcB
Peptide, recombinant protein	Endoglycosidase-H	New England Biolabs	Catalog #P0702S	https://www.neb.com/products/p0702-endo-h#Product%20Information

### Protein purification

HEK293S GnTI^-/-^ and HEK293F cell lines were used for protein expression. Both cells lines were authenticated using STR profiling. Mycoplasma tests were performed using the MycoProbe kit from R and D Systems and the samples were negative for contamination.

#### 426c core^†^-VRC01_GL_

426c core^†^-VRC01_GL_ was expressed using HEK293S GnTI^-/-^ cells . Cells were cultured in suspension and transfected with equal parts of 426c G459C core, VRC01_GL-_A60C_HC_, and VRC01_GL_ light chain plasmids (500 µg total/L) using 293 Free Transfection Reagent (Novagen). After 6 days, cells were centrifuged at 4,500 rpm for 20 min and supernatant was filter-sterilized. A His-tag on the Fab heavy chain was utilized for purification by suspending His60 Ni-Superflow Resin (Takata) in the supernatant at 4°C overnight. The Ni resin was washed with a solution of 150 mM NaCl, 20 mM Tris pH 8.0, 20 mM Imidazole pH 7.0 and eluted with a solution of 300 mM NaCl, 50 mM Tris pH 8.0, 250 mM Imidazole pH 7.0. The sample was further purified by Size-exclusion chromatography (SEC) using a HiLoad 16/600 Superdex 200 pg (GE) column removing non-specific proteins and excess Fab. Fractions containing the complex were concentrated and treated with an excess of EndoH for one hour at 37°C. The complex was then rerun over an SEC S200 column and concentrated to ~10 mg/mL for crystallization trials.

#### 426c DS-SOSIP D3^†^-VRC01_GL_

HEK293S GnTI^-/-^ cells were transfected with 426c DS-SOSIP D3^†^, VRC01_GL_-A60C heavy chain, VRC01_GL_ light chain and furin plasmids at a ratio of 3:1:1:1, as described above and previously (Stewart-Jones et al., 2016). Complexes were purified by the His-tag on the VRC01_GL_ fragment as described above. Complexes were further purified on SEC as previously described and the peak containing both SOSIP and VRCO1_GL_ were concentrated for cryoEM.

#### 426c DS-SOSIP variants

426c DS-SOSIP D3 (non-crosslinked G459 variant with C-terminal strep-his tag) was expressed using HEK293F cells by co-transfection of 426c DS.SOSIP D3 and furin plasmids at a 5:1 ratio. The cells were not tested for mycoplasma contamination. 426c DS-SOSIP D3 was purified first by Ni-affinity and then by Streptactin-affinity, followed by enzymatic digestion and separation of the cleaved tag from the trimer by SEC using a HiLoad 16/600 Superdex 200 pg (GE).

#### 426c core constructs

All 426c core constructs were expressed by the transfection protocol described above. Agarose Bound Galanthus Nivalis Lectin (Vector) was used to separate the cores from the supernatant. The resin was washed with 20 mM Tris pH 7.5, 100 mM NaCl, 1 mM EDTA and elution used a buffer containing 20 mM Tris pH 7.5, 100 mM NaCl, 1 mM EDTA, and 1M Methyl α-D-mannopyranoside. Samples were further purified by SEC using a HiLoad 16/600 Superdex 200 pg (GE) column.

#### Antibodies

All antibodies were expressed by the transfection protocol described above using equal ratios of heavy and light chain encoding plasmids. Protein A Agarose (Pierce) resin was used to separate IgG from the supernatant. Protein A beads were washed with phosphate buffer saline (PBS) and elution used a commercially available IgG elution buffer at pH 2.0 (Pierce). Samples were buffer exchanged into PBS.

### Biolayer interferometry

BLI assays were performed with an Octet Red 96 instrument (ForteBio, Inc, Menlo Park, CA) at 29°C with shaking at 500 r.p.m. All measurements were corrected by subtracting the background signal obtained from duplicate traces generated with an irrelevant negative control IgG or Fab. For standard BLI assays, IgGs were immobilized on anti-AHC biosensors (at 20 µg/ml in PBS), or Fabs on anti-human Fab-CH1 (FAB2G, ForteBio) biosensors (at 40 µg/ in PBS), for 240 s. Sensors were then incubated for 1 min in kinetic buffer (KB: 1X PBS, 0.01% BSA, 0.02% Tween 20% and 0.005% NaN_3_) to establish the baseline signal (nm shift). Antibody-loaded sensors were then immersed into solutions of purified recombinant samples for kinetic analysis. Analyses of DS-SOSIP trimers and 426c core constructs was performed by BLI using VRC01_GL_ IgG and an extensive dilution series to determine accurate K_D_ estimates. Samples expressed in the presence of 100 µM kifunensin and EndoH-digested were first buffer exchanged into PBS prior to dilution and kinetic analyses. Curve fitting to determine relative apparent antibody affinities for the samples was performed using a 1:1 binding model and the ForteBio data analysis software. Mean k_on_, k_off_, and K_D_ values were determined by averaging all binding curves within a dilution series having R^2^ values of greater than 95% confidence level.

### Crystallization

Crystallization conditions for the 426c core^†^-VRC01_GL_ were screened using a Mosquito (ttplabtech)-dispensing robot. Screening was done with Rigaku Wizard Precipitant Synergy block no. 2, Molecular Dimensions Proplex screen HT-96, and Hampton Research Crystal Screen HT using the vapor diffusion method. Initial crystals were further optimized with Hampton Research Additive Screen to grow large and well-diffracting crystals. Final crystals were grown in a solution of 0.09M MgCl_2_, 0.09M Na-Citrate pH 5.0, 13.5% PEG 4000, 0.1M LiCl_2_. Crystals were cryoprotected in solutions containing 30% molar excess of their original reagents and 20% glycerol. Crystals diffracted to 2.3 Å. Data was collected at ALS 5.0.2 and processed using HKL2000 (Otwinowski and Minor, 1997).

### Structure solution and refinement

The structure of 426c core^†^-VRC01_GL_ Fab was solved through molecular replacement using Phaser in CCP4 (Collaborative Computational Project, Number 4, 1994). The structure was further refined with COOT (Emsley and Cowtan, 2004) and Phenix (Adams et al., 2010). The refinement statistics are summarized in Table 1.

### Negative-stain EM sample preparation

All 426c DS-SOSIP constructs in this study (3 µL) were negatively stained at a final concentration of 0.008 mg/mL using Gilder Grids overlaid with a thin layer of carbon and 2% uranyl formate as previously described (Veesler et al., 2014).

### Negative-Stain EM data collection and processing

Data were collected on an FEI Technai 12 Spirit 120kV electron microscope equipped with a Gatan Ultrascan 4000 CCD camera. A total of 150–300 images were collected per sample by using a random defocus range of 1.1–2.0 µm with a total exposure of 45 e−/A^2^. Data were automatically acquired using Leginon (Suloway et al., 2005), and data processing was carried out using Appion (Lander et al., 2009). The parameters of the contrast transfer function (CTF) were estimated using CTFFIND4 (Mindell and Grigorieff, 2003), and particles were picked in a reference-free manner using DoG picker (Voss et al., 2009). Particles were extracted with a binning factor of 2 after correcting for the effect of the CTF by flipping the phases of each micrograph with EMAN 1.9 (Ludtke et al., 1999). The 426c DS-SOSIP D3^†^-VRC01_GL_ stack was pre-processed in RELION/2.1 (Kimanius et al., 2016; Scheres, 2012b; Scheres, 2012a) with an additional binning factor of 2 applied, resulting in a final pixel size of 6.4 Å. Resulting particles were sorted by reference-free 2D classification over 25 iterations. The best particles were chosen for 3D classification into six classes using RELION/2.1 (Kimanius et al., 2016). C3 symmetry was applied for 426c DS-SOSIP D3^†^-VRC01_GL_, with the best 3D classes refined further in RELION/2.1 (Kimanius et al., 2016) using the gold-standard approach.

### CryoEM sample preparation

We applied 2 μL of 0.7 mg/mL of DS-SOSIP D3^†^-VRC01_GL_ in 10 mM HEPES pH 7.5, 50 mM NaCl, 0.085 mM dodecyl-maltoside to glow-discharged C-flat CF-1.2/1.3–4 C-T-grids. Vitrification was performed by using an FEI Vitrobot Mark IV, using a blot time of 6 s at a temperature of 22°C and 100% humidity.

### CryoEM data collection

Data collection was performed automatically using Leginon (Suloway et al., 2005) to control an FEI Titan Krios Electron Microscope equipped with a Gatan Quantum GIF energy filter and a K2 Summit direct electron detector(Li et al., 2013) operating in electron-counting mode spanning a random defocus range between 2.0 and 3.5 μm. Approximately 2000 micrographs were collected with a pixel size of 1.36 Å at a dose rate of 8 counts per pixel per second and 15 s acquisition time (0.2 frame per second), yielding a final measured dose of 43 e−/Å^2^ per movie.

### CryoEM data processing

Alignment of movie frames was carried out using MotionCor2 (Zheng et al., 2017) with a B-factor of −100 Å^2^ and an applied dose-weighting scheme of 0.95 electrons/Å^2^/frame. Omission of low-quality micrographs left a total of 1724 micrographs for downstream data processing. ~567,000 particles were picked in a reference-free manner using DoG picker (Voss et al., 2009). Global defocus and astigmatism were estimated using GCTF (Zhang, 2016) on the non-dose weighted aligned sums. Dose-weighted particles were binned to a final pixel size of 5.44 Å for an initial round of 2D classification using RELION/2.1 (Kimanius et al., 2016). 200,000 selected particles were re-centered, re-extracted, and unbinned to a final pixel size of 1.36 Å and subjected to 3D classification with RELION/2.1 (Kimanius et al., 2016) using the 30 Å low-pass filtered initial model generated from the DS-SOSIP D3^†^-VRC01_GL_ negative-stain dataset. Out of the eight resulting classes, five classes contained well defined secondary structure elements and three bound Fabs. These classes were low-pass filtered to 20 Å and the best-resolved class was used as an initial model during 3D refinement using C3 symmetry. Refined angles for all particles were subsequently imported into FREALIGN (Grigorieff, 2016; Lyumkis et al., 2013a) and further refined with an applied particle weighting scheme. An additional iteration of refinement was performed by adjusting only the X/Y shifts. This refinement scheme resulted in a final estimated resolution of 3.8 Å for the three-Fab complex. The VRC01_GL_ constant domains of the Fab were masked out during the final rounds of refinement and omitted from the final model due to the inherent flexibility of the elbow region (Stanfield et al., 2006). This same strategy was used for 3D classes of DS-SOSIP D3^†^-VRC01_GL_ containing only two Fabs (C1 symmetry), leading to a final estimated resolution of 4.8 Å. Reported resolutions are based on the gold-standard FSC = 0.143 criterion. Local resolution estimates were generated using the ResMap software (Kucukelbir et al., 2014).

### Model building and refinement

We selected a clade A HIV-1 BG505 SOSIP.664 trimer (Stewart-Jones et al., 2016) and the 426c.TM1deltaV1-V3gp120 in complex with germline NIH46-46 (Scharf et al., 2016) as initial reference models for building 426c DS-SOSIP D3^†^-VRC01_GL_. This model was manually trimmed and edited using Coot (Emsley and Cowtan, 2004; Emsley et al., 2010) and RosettaES (Frenz et al., 2017). We then further refined the structure in Rosetta using density-guided protocols (Wang et al., 2016) for the 3.8 Å resolution C3 reconstruction. This process was repeated iteratively until convergence and high agreement with the map was achieved. The Fab constant domains were masked out during refinement and omitted from the final model. Following refinement of protein coordinates, identified *N*-linked glycans were manually docked into their corresponding density and refined using Rosetta (DiMaio et al., 2011; Frenz et al., 2018). Multiple rounds of minimization were performed on the complete glycoprotein model and manually inspected for errors. Throughout this process, we applied strict non-crystallographic symmetry constraints in Rosetta (DiMaio et al., 2011). The 4.8 Å asymmetric 426c DS-SOSIP D3^†^-VRC01_GL_ structure bound to only two Fabs was obtained by removing one of the Fabs bound to the aforementioned model and was rigid-body docked into the 2 Fab-bound map using UCSF Chimera.. Mannose rings not supported by density in this map were manually trimmed. Final model quality was analyzed using Molprobity (Chen et al., 2010) and EM ringer (Barad et al., 2015). All figures were generated with UCSF Chimera (Pettersen et al., 2004).

### VRC01_GL_-based Immunoprecipitation

Purified recombinant VRC01_GL_ IgG was covalently coupled to Dnyabeads MyOne Tosylactivated beads (Life Technologies), and immunoprecipitation using magnetic separation was carried out according to the manufacturer’s protocol. 5 mg of 426c core produced using HEK293S GnTI^-/-^ cells were incubated with 100 μg of VRC01_GL_-beads for 15 min (first bind), after which the beads were removed and unbound material (flow through) was incubated with a second fresh 100 μg aliquot of VRC01_GL_-beads for an additional 15 min (second bind). VRC01_GL_-beads from first and second binding were washed 3x before acidic elution and pH neutralization of affinity-purified samples. Unbound material was further depleted by incubation with a third 100 μg of VRC01_GL_-beads, which were removed, before analysis. Samples of the original input 426c core, and VRC01_GL_-bound and unbound fractions were resolved by SDS gel electrophoresis under reducing conditions and the remainder subjected to LC-MS/MS analysis, as described above.

### Mass spectrometry

For analysis of *N*-linked glycosylation profiles, an estimated 250 pmol of each HIV-1 426c-based construct analyzed in this paper was denatured, reduced, and alkylated by dilution to 5 μM in 50 μL of buffer containing 100 mM Tris (pH 8.5), 10 mM Tris(2-carboxyethylphosphine (TCEP), 40 mM iodoacetamide or 40 mM iodoacetic acid, and 2% (wt/vol) sodium deoxycholate. Samples were first heated to 95°C for 10 min and then incubated for an additional 30 min at room temperature in the dark. The samples were digest with trypsin (Sigma Aldrich), by diluting 20 μL of sample to total volume of 100 μL 50 mM ammonium bicarbonate (pH 8.5). Protease was added to the samples in a ratio of 1:75 by weight and left to incubate at 37°C overnight. After digestion, 2 μL of formic acid was added to the samples to precipitate the sodium deoxycholate from the solution. After centrifugation at 17,000 × g for 25 min, 85 μL of the supernatant was collected and centrifuged again at 17,000 × g for 5 min to ensure removal of any residual precipitated deoxycholate. 80 μL of this supernatant was collected. For each sample, 8 μL was injected on a Thermo Scientific Orbitrap Fusion Tribrid mass spectrometer. A 35 cm analytical column and a 3 cm trap column filled with ReproSil-Pur C18AQ 5 μM (Dr. Maisch) beads were used. Nanospray LC-MS/MS was used to separate peptides over a 90 min gradient from 5% to 30% acetonitrile with 0.1% formic acid. A positive spray voltage of 2100 was used with an ion transfer tube temperature of 350°C. An electron-transfer/higher-energy collision dissociation ion-fragmentation scheme (Frese et al., 2013) was used with calibrated charge-dependent entity-type definition (ETD) parameters and supplemental higher-energy collision dissociation energy of 0.15. A resolution setting of 120,000 with an AGC target of 2 × 10^5^ was used for MS1, and a resolution setting of 30,000 with an AGC target of 1 × 10^5^ was used for MS2. Data were searched with the Protein Metrics Byonic software (Bern et al., 2012), using a small custom database of recombinant protein sequences including the proteases used to prepare the glycopeptides. Reverse decoy sequences were also included in the search. Specificity of the search was set to C-terminal cleavage at R/K (trypsin), allowing up to two missed cleavages, with EthcD fragmentation (b/y- and c/z-type ions). We used a precursor mass and product mass tolerance of 12 ppm and 24 ppm, respectively. Carbamidomethylation of cysteines was set as fixed modification, carbamidomethylation of the lysines and N-terminal amines were set as variable modifications, methionine oxidation as variable modification, pyroglutamate identification was set for both N-terminal glutamines and glutamates as a variable modification, and a concatenated N-linked glycan database (derived from the four software-included databases) was used to identify glycopeptides. All analyzed glycopeptide hits were manually inspected to ensure for quality and accuracy. Semi-quantitative LC-MS/MS of VRC01-based immunoprecipitation experiments were performed using Skyline (MacLean et al., 2010) with peak integration and LC-MS/MS searches imported from Byonic. Missed cleavages and post-translational modifications listed above for qualitative LC-MS/MS searches were included in the quantification of glycopeptides. All MS1 peak areas used for integration were manually inspected to ensure for quality and accuracy. Unbound fractions from two experimental replicates were pooled and injected as two technical replicates, whereas each ‘bound’ fraction (first bind and second bind) were performed as two experimental and two technical replicates each.

## Additional files

Supplementary file 1. BLI kinetics parameters of various 426c core constructs expressed using HEK293F cells.

Supplementary file 2. RMSD Table comparing unliganded and liganded VRC01_GL_ CDRL1 loop conformations.

Supplementary file 3. BLI kinetics parameters of various 426c core constructs expressed using GnTI^-/-^ cells.

Transparent reporting form

### Data availability

Mass spectrometry data have been deposited to the PRIDE archive under accession number PXD011494. CryoEM maps are available for download from the EMDB under accession numbers EMD-9294 (426cDS-SOSIP D3^†^-VRC01_GL_, 3 Fabs, sharpened), EMD-9295 (426cDS-SOSIP D3^†^-VRC01_GL_, 3 Fabs, unsharpened), EMD-9304 (426cDS-SOSIP D3^†^-VRC01_GL_, 2 fabs, unsharpened), and EMD-9303 (426cDS-SOSIP D3^†^-VRC01_GL_, 2 fabs, sharpened). Structures have been deposited to the PDB under accession numbers PDB 6MYY (426cDS-SOSIP D3^†^-VRC01_GL_, 3 Fabs]), PDB-6MZJ (426cDS-SOSIP D3^†^-VRC01_GL_, 2 fabs), and 6MFT (426c core^†^-VRC01_GL_)

The following datasets were generated:

AndrewJ BorstConnorE WeidleMatthewD GrayBrandonFrenzJoostSnijderMGordon JoyceIvelinS GeorgievGuillaumeBE Stewart-JonesPeterD KwongAndrewT McGuireFrankDiMaioLeonidasStamatatosMariePanceraDavidVeesler2018Germline VRC01 antibody recognition of a modified clade C HIV-1 envelope trimer, 3 Fabs bound, sharpened mapEMBL-EBI Protein Data BankEMD-9294

AndrewJ BorstConnorE WeidleMatthewD GrayBrandonFrenzJoostSnijderMGordon JoyceIvelinS GeorgievGuillaumeBE Stewart-JonesPeterD KwongAndrewT McGuireFrankDiMaioLeonidasStamatatosMariePanceraDavidVeesler2018Germline VRC01 antibody recognition of a modified clade C HIV-1 envelope trimer, 2 Fabs bound, unsharpened mapEMBL-EBI Protein Data BankEMD-9304

AndrewJ BorstConnorE WeidleMatthewD GrayBrandonFrenzJoostSnijderMGordon JoyceIvelinS GeorgievGuillaumeBE Stewart-JonesPeterD KwongAndrewT McGuireFrankDiMaioLeonidasStamatatosMariePanceraDavidVeesler2018Germline VRC01 antibody recognition of a modified clade C HIV-1 envelope trimer, 2 Fabs bound, sharpened mapRSCB Protein Data Bank6MZJ

AndrewJ BorstConnorE WeidleMatthewD GrayBrandonFrenzJoostSnijderMGordon JoyceIvelinS GeorgievGuillaumeBE Stewart-JonesPeterD KwongAndrewT McGuireFrankDiMaioLeonidasStamatatosMariePanceraDavidVeesler2018Germline VRC01 antibody recognition of a modified clade C HIV-1 envelope trimer, 2 Fabs bound, sharpened mapEMBL-EBI Protein Data BankEMD-9303

AndrewJ. BorstConnorE. WeidleMatthewD. GrayBrandonFrenzJoostSnijderM.Gordon JoyceIvelinS. GeorgievGuillaumeB.E. Stewart-JonesPeterD. KwongAndrewT. McGuireFrankDiMaioLeonidasStamatatosMariePanceraDavidVeesler2018Germline VRC01 antibody recognition of a modified clade C HIV-1 envelope trimer, 3 Fabs bound, sharpened mapRCSB Protein Data Bank6MYY

AndrewJ BorstConnorE WeidleMatthewD GrayBrandonFrenzJoostSnijderMGordon JoyceIvelinS GeorgievGuillaumeBE Stewart-JonesPeterD KwongAndrewT McGuireFrankDiMaioLeonidasStamatatosMariePanceraDavidVeesler2018Germline VRC01 antibody recognition of a modified clade C HIV-1 envelope trimer, 3 Fabs bound, unsharpened mapEMBL-EBI Protein Data BankEMD-9294

AndrewJ. BorstConnorE. WeidleMatthewD. GrayBrandonFrenzJoostSnijderM.Gordon JoyceIvelinS. GeorgievGuillaumeB.E. Stewart-JonesPeterD. KwongAndrewT. McGuireFrankDiMaioLeonidasStamatatosMariePanceraDavidVeesler2018Recombinant HIV-1 426c Env Tryptic Digest Glycan Identifications and Semi-QuantificationEBI PRIDEPXD011494

AndrewJ BorstConnorE WeidleMatthewD GrayBrandonFrenzJoostSnijderMGordon JoyceIvelinS GeorgievGuillaumeBE Stewart-JonesPeterD KwongAndrewT McGuireFrankDiMaioLeonidasStamatatosMariePanceraDavidVeesler2018Crystal structure of glycosylated 426c HIV-1 gp120 core G459C in complex with glVRC01 A60C heavy chainRCSB Protein Data Bank6MFT
