# Supplementary material for: Germline VRC01 antibody recognition of a modified clade C HIV-1 envelope trimer and a glycosylated HIV-1 gp120 core
Source: eLife. 2018 Nov 7;7:e37688. doi: 10.7554/eLife.37688 (PMC6237438; doi:10.7554/eLife.37688)
Supplement: Supplementary file 1. [file elife-37688-supp1.docx]

|  |  |  | Average Kinetic Values | | |  |  |
| --- | --- | --- | --- | --- | --- | --- | --- |
| VRC01_GL_ Fab vs 426c core (293) | KD (M) | KD Error | kon(1/Ms) | kon Error | kdis(1/s) | kdis Error | Full R^2^ |
| 426c core S278A | 1.89E-06 | 1.04E-07 | 3.02E+03 | 1.08E+02 | 4.81E-03 | 9.06E-05 | 0.9972 |
| 426c core S278A/T462A | 2.50E-06 | 1.79E-07 | 3.40E+03 | 1.74E+02 | 6.63E-03 | 1.53E-04 | 0.9635 |
| 426c core S278A/T465A | 8.90E-06 | 5.19E-07 | 2.92E+03 | 1.62E+02 | 1.36E-02 | 2.23E-04 | 0.9626 |
| 426c core S278A/T462A/T465A | 8.61E-06 | 6.88E-07 | 5.49E+03 | 4.86E+02 | 2.58E-02 | 6.00E-04 | 0.9460 |
|  |  |  |  |  |  |  |  |
|  |  |  |  |  |  |  |  |
