# Supplementary material for: Germline VRC01 antibody recognition of a modified clade C HIV-1 envelope trimer and a glycosylated HIV-1 gp120 core
Source: eLife. 2018 Nov 7;7:e37688. doi: 10.7554/eLife.37688 (PMC6237438; doi:10.7554/eLife.37688)
Supplement: Supplementary file 2. [file elife-37688-supp2.docx]

| Cα RMSD for residues 26-32  (Atoms) | WT426c core - VRC01_GL_  chain L | WT426c core - VRC01_GL_ chain B |
| --- | --- | --- |
| 4JPI (unliganded VRC01_GL_)  chain L | 1.637  (2.687) | 1.759  (2.598) |
| 4JPI (unliganded VRC01_GL_)  chain B | 0.379  (1.271) | 0.667  (1.530) |
| 4JPK  chain L | 0.909  (1.731) | 1.066  (1.879) |
| 5IGX  chain L | 1.601  (2.600) | 1.700  (2.446) |
