# Supplementary material for: Germline VRC01 antibody recognition of a modified clade C HIV-1 envelope trimer and a glycosylated HIV-1 gp120 core
Source: eLife. 2018 Nov 7;7:e37688. doi: 10.7554/eLife.37688 (PMC6237438; doi:10.7554/eLife.37688)
Supplement: Supplementary file 3. [file elife-37688-supp3.docx]

|  |  |  | Average Kinetic Values | | |  |  |
| --- | --- | --- | --- | --- | --- | --- | --- |
| VRC01_GL_ IgG vs 426c core (GNTi^-/-^) | KD (M) | KD Error | kon(1/Ms) | kon Error | kdis(1/s) | kdis Error | Full R^2^ |
| 426c core | 2.25E-06 | 4.18E-08 | 1.26E+03 | 2.18E+01 | 2.69E-03 | 1.62E-05 | 0.9804 |
| 426c core +kif | N/D | N/D | N/D | N/D | N/D | N/D | N/D |
| 426c core (100 µM kifunensin) +EndoH | 2.45E-07 | 6.03E-09 | 1.17E+04 | 2.76E+02 | 2.51E-03 | 2.23E-05 | 0.9547 |
| 426c S278A core | 2.04E-07 | 4.95E-09 | 3.58E+04 | 7.95E+02 | 5.31E-03 | 3.94E-05 | 0.9804 |
